# Multidimensional tumor-blood profiling uncovers systemic lymphocyte-monocyte imbalance in pituitary neuroendocrine tumors

**DOI:** 10.1038/s41392-025-02489-0

**Published:** 2025-11-18

**Authors:** Yuting Dai, Shaojian Lin, Junchen Wu, Shuangshuang Yang, Yang Lu, Xiaobin Wang, Jun Li, Linfeng Zhao, Desheng Chen, Bo Zhang, Yijun Cheng, Hong Yao, Fan Zhang, Min Xu, Qiang Wang, Xiaojing Lin, Kunjin Chen, Zhen Tian, Xingyan Liu, Pascal Roy, Hai Fang, Gang Lv, Tong Yin, Yun Tan, Bo Jiao, Shengyue Wang, Li Xue, Youqiong Ye, Saijuan Chen, Zhe Bao Wu

**Affiliations:** 1 Department of Neurosurgery, Center of Pituitary Tumor, Ruijin Hospital, Shanghai Jiao Tong University School of Medicine, Shanghai, China; 2 Shanghai Institute of Hematology, State Key Laboratory of Medical Genomics, National Research Center for Translational Medicine at Shanghai, Research Unit of Hematologic Malignancies Genomics and Translational Research of Chinese Academy of Medical Sciences, Ruijin Hospital, Shanghai Jiao Tong University School of Medicine, Shanghai, China; 3 Department of Neurosurgery, The First Affiliated Hospital of Wenzhou Medical University, Wenzhou, China; 4 Department of Neurosurgery, The First Affiliated Hospital of Henan University, Henan, China; 5 Center for Immune-Related Diseases at Shanghai Institute of Immunology, Ruijin Hospital, Shanghai Jiao Tong University School of Medicine, Shanghai, China; 6 Shanghai Institute of Immunology, Shanghai Jiao Tong University School of Medicine, Shanghai, China; 7 Université Claude Bernard Lyon 1 Université de Lyon, Lyon, France; 8 Shanghai Institute of Immunology, Department of Immunology and Microbiology at Basic Medical College, Shanghai Jiao Tong University School of Medicine, Shanghai, China

**Keywords:** Endocrine cancer, Tumour immunology

## Abstract

Pituitary neuroendocrine tumors (PitNETs) are pathologically characterized by dysregulation of neuroendocrine function and systemic disruption of hormonal homeostasis, yet their regulatory effects on peripheral immune networks remain poorly characterized. Here, we systematically analyzed bulk RNA sequencing (RNA‑seq) from 883 PitNET tumors, 108 PitNET‑associated peripheral blood mononuclear cells (PBMC) samples, and 175 healthy PBMC controls, combined with 69 single‑cell RNA sequencing (scRNA-seq) samples covering tumors, normal pituitaries, as well as tumor‑derived and normal PBMCs. We identified a systemic immune disequilibrium in PitNET patients, characterized by increased circulating lymphocyte proportions, accompanied by upregulated cytokine-receptor interaction signatures. Notably, tumor resection reversed this imbalance, as supported by the normalization of monocyte and neutrophil counts, validated by flow cytometry and routine blood data from 600 samples (200 healthy controls and 200 PitNET patients with paired pre- and post-surgery follow‑up). Trajectory analysis identified terminally differentiated, secretory-specialized cell populations with lineage-specific hormone and cytokine hypersecretion. Ligand-receptor inference suggested these tumor-derived factors potentially engage circulating immune cell receptors. A random‑forest classifier based on PBMC transcriptomes distinguished PitNET subtypes, underscoring the diagnostic potential of peripheral immune signatures. Furthermore, in an estrogen-induced rat model, elevated PRL level coincided with the same peripheral immune skewing. Overall, our work provides a valuable resource and demonstrates PitNETs can be systemic immune modulators, where intrinsic hormone secretory activity and monocyte-lymphocyte imbalance collectively drive peripheral immune dysfunction.

## Introduction

Pituitary neuroendocrine tumors (PitNETs), originating from hormone-secreting cells of the anterior pituitary, represent a unique class of primary intracranial neoplasms that possess both endocrine and oncological significance.^1–3^ Epidemiological data estimates their incidence at approximately 4.54 cases per 100,000 individuals each year, accounting for 17.2% of all primary brain tumors, positioning them as the second most common intracranial neoplasm after meningiomas.^4^ The 2022 World Health Organization (WHO) classification refines the molecular stratification of PitNETs into three transcription factor-driven lineages: PIT1-lineage tumors (lactotrophs, thyrotrophs, somatotrophs), TPIT-lineage (corticotrophs), and SF1-lineage (gonadotrophs), with a very small subset of null cell tumors lacking lineage-specific markers.^5^ Clinically, PitNETs exhibit dual characteristics. On one hand, functional PitNETs excessively secrete hormones such as prolactin (PRL), adrenocorticotropic hormone (ACTH), and growth hormone (GH),^6–9^ leading to systemic endocrine disorders like infertility induced by prolactinoma, metabolic dysregulation associated with Cushing’s disease, and organomegaly linked to acromegaly.^10,11^ On the other hand, non-functional PitNETs, accounting for approximately 15–35% of cases (predominantly within the SF1-lineage), often remain undetected until significant mass effects occur, resulting in complications such as bitemporal hemianopsia or cranial nerve palsies. Consequently, PitNETs extend beyond mere intracranial lesions to serve as systemic disease modifiers, necessitating a broader perspective on their extra-pituitary impact.^12^

PitNETs present a complex immune microenvironment that significantly impacts tumor behavior, therapeutic response, and prognosis. Despite its generally low immune cell infiltration dominated by macrophages and T cells,^13–15^ specific immune interactions remain evident. For example, CX3CR1^+^ macrophages in SF1-lineage tumors play a nuanced role of immune cells in tumor apoptosis via INHBA-ACVR1B signaling.^13^ CD8^+^ T cell infiltration is notably prevalent in growth hormone-secreting adenomas (GHomas), correlating negatively with tumor aggressiveness and predicting better responses to somatostatin analogs.^16,17^ Conversely, tumor-infiltrating T cells in SF1-lineage gonadotrophs promote immune evasion through the release of immunosuppressive cytokines such as IL-10^18^. High macrophage infiltration is linked to larger, invasive tumors, while reduced NK cell numbers suggest diminished immune surveillance in aggressive PitNETs.^16,19^ Immune profiling, particularly of CD8^+^ T cells, PD-L1 expression and B cell infiltration can predict immunotherapy efficacy.^20,21^ Among them, CD8⁺ effector and memory T cells exhibit strong cytotoxicity through IFN-γ and TNF-α release, and their higher abundance has been linked to reduced invasiveness and more favorable clinical outcomes.^22^ Moreover, tumor-derived cytokines and chemokines including CXCL1, CCL2, CCL5, and CXCL12 shape the tumor microenvironment by recruiting and polarizing immune cells, modulating angiogenesis, and influencing epithelial-mesenchymal transition.^23,24^ These findings highlight the potential for immune-targeted therapies, such as PD-1/PD-L1 inhibitors and CAR-T cell therapies, particularly for invasive non-functional PitNETs.^25–27^ Furthermore, as endocrine-active lesions, PitNETs may exert broader systemic immune modulation.

Peripheral blood mononuclear cells (PBMCs), the mobile sentinels of systemic immunity, could thus serve as mediators and biomarkers of this tumor-host interaction. The dynamic changes in PBMCs have demonstrated prognostic value in various cancer immunotherapies. For instance, in hepatocellular carcinoma patients treated with pembrolizumab, responders exhibited an immune profile shift towards cytotoxic CD8^+^ T cells, whereas non-responders showed an increase in CD14^+^ and CD16^+^ monocytes.^28^ Additionally, the frequency of CD4^+^CD25^+^FOXP3^+^ regulatory T cells (Tregs) in PBMCs has been correlated with immunotherapy outcomes, with patients having lower baseline Treg levels experiencing longer survival.^29^ PBMC-derived immune cells can exert anti-tumor activity through four mechanisms: direct cytotoxicity,^30^ immune checkpoint modulation,^31^ cytokine production^32^ and the bystander effect.^33^ However, whether PitNETs similarly reshape PBMC dynamics remains uncharted. Integrating knowledge from both tumor tissues and peripheral immune components could therefore furnish a more holistic understanding of PitNET progression and help identify new biomarkers for disease monitoring.

**Fig. 1:**
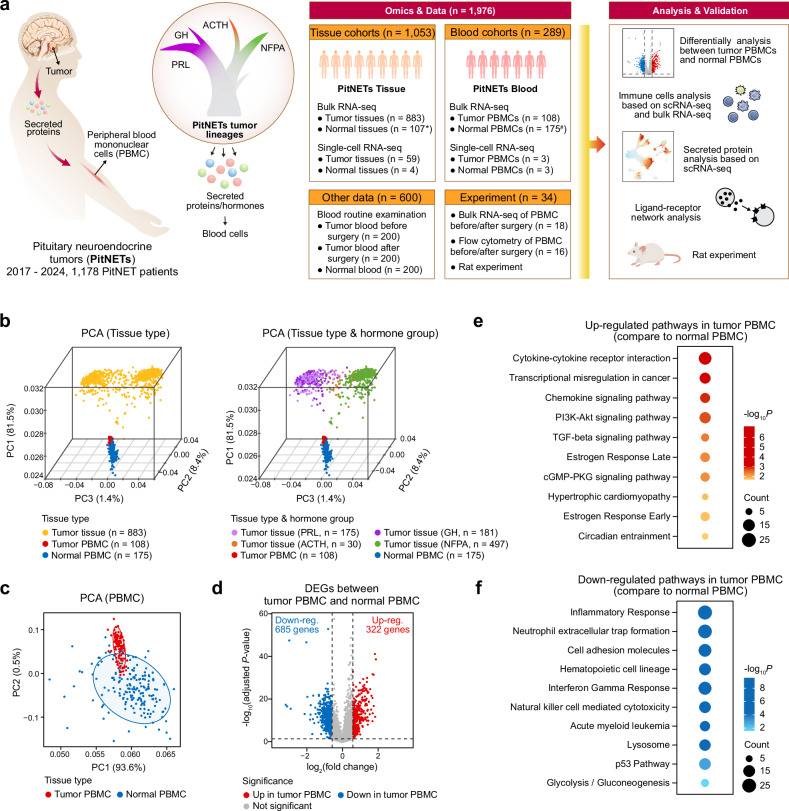
Study design overview and pathway hallmarks in PitNET PBMCs. **a** Schematic representation of the overall study framework, integrating large-scale transcriptomic and single-cell datasets from PitNET tumor tissues, PitNET PBMC and normal PBMC samples. ^*^The gene expression data from normal pituitary tissues (*n* = 107) were obtained from the genotype-tissue expression database (GTEx)^83^ of the Broad Institute. ^#^For normal PBMCs (*n* = 175), nine of them were in-house generated, and others were obtained from public database. **b** Principal Component Analysis (PCA) plots using protein-coding genes. In the left panel, samples are colored by tissue type: yellow indicates tumor tissues, red denotes PitNET PBMCs, and blue corresponds to normal PBMCs. In the right panel, tumor tissues are further classified by hormone groups (PRL, GH, ACTH, and NFPA). The % value indicates the explained variance. **c** PCA plot focusing exclusively on PBMCs, illustrating the separation between PitNET PBMCs (red) and normal PBMCs (blue). **d** Volcano plot showing DEGs between PitNET PBMCs and normal PBMCs. Significantly upregulated genes (red) were identified by adjusted *P-*value < 0.05 and log_2_(fold change) > 0.58 (fold change > 1.5), whereas significantly downregulated genes (blue) satisfy adjusted *P-*value < 0.05 and log_2_(fold change) < -0.58. Other genes (gray) are considered not significant. Statistical analyses were performed using the limma^36^ R package, and *P-*values were adjusted with Benjamini-Hochberg correction. Two-sided *P-*values were used. Dot plots highlighting significantly enriched pathways using upregulated (**e**) and downregulated (**f**) DEGs in PitNET PBMCs compared with normal PBMCs. Each dot represents a pathway calculated via Enrichr,^81^ colored by enrichment *P-*values (calculated by Enrichr) and sized according to gene count. Red dots mark pathways significantly enriched for upregulated genes, while blue dots mark those enriched for downregulated genes. Two-sided *P-*values were used

To address this gap, we conducted a multidimensional study coupling tumor tissue profiles with peripheral blood immune data. By leveraging bulk RNA sequencing (RNA-seq) on 883 tumors, 108 PitNET PBMC samples, and 175 healthy controls; single-cell RNA sequencing (scRNA-seq) on 59 tumor samples, 4 normal pituitaries, 3 PitNET PBMC samples, and 3 PBMC samples from healthy individuals; and clinical validation via flow cytometry, blood routine examinations, and postoperative PBMC tracking (Fig. 1a), we mapped the systemic immune landscape of PitNETs. Our analyses reveal that tumor lineage-specific secretory programs interact with corresponding receptors on PBMCs to perturb the balance of lymphoid and myeloid cell populations, which can be reversed by tumor resection. Complementing these findings with estrogen-driven rat models, we identify PitNETs as key modulators of a tumor-peripheral immune axis, wherein secretory ligands (e.g., lineage-specific hormones or chemokines) directly influence circulating immune cells. This work redefines PitNETs as immunomodulatory entities and positions PBMCs as dynamic biomarkers, offering a framework for therapeutic strategies targeting hormone-immune crosstalk.

## Results

### Patients with PitNETs

We conducted a prospective observational study involving two Chinese cohorts, encompassing 1178 PitNET patients who underwent surgical resection at Ruijin Hospital (denoted as the ‘Ruijin cohort’) and Beijing Tiantan Hospital (denoted as the ‘Tiantan cohort’) from 2017 to 2024 (Supplementary Table 1a–e). A total of 883 tumor tissue samples (566 from the Ruijin cohort and 317 from the Tiantan cohort) underwent bulk RNA-seq. Batch effects were corrected using ComBat^34^ (Supplementary Fig. 1a, Supplementary Table 1a). Subsequently, ConsensusClusterPlus^35^ was used to determine the optimal number of clusters of tumor tissues (Supplementary Fig. 1b, Supplementary Fig. 1c), leading to the identification of six distinct subgroups (G1 to G6) of PitNETs through unsupervised clustering based on the top 5% of genes with the highest variance^8^ (Supplementary Fig. 1d). The distribution of samples was consistent with our previous reports.^8,13^ Specifically, G1, G2, and G3 displayed elevated *POU1F1* (PIT1-lineage, 41.2% of total cases), G4 and G5 were characterized by high *TBX19* (TPIT-lineage, 19.8%), and G6 showed increased *NR5A1* (SF1-lineage, 39.0%) (Supplementary Fig. 1e). Based on hormone secretion status, tumors were assigned to four hormone-defined groups (PRL, GH, ACTH or clinical NFPA) supported by WHO histological classification and RNA-seq subtype (Supplementary Table 1a, 1b). Principal component analysis (PCA) further verified the separation of these three major lineages and four hormone groups (Supplementary Fig. 1f).

Next, we performed PCA on 19,353 protein-coding genes across three sample types, including 883 PitNET tumor tissues, 108 PitNET PBMCs, and 175 normal PBMCs. As a result, significant difference was shown between tumor tissue samples and both types of PBMC samples after adjusting for batch effects (Fig. 1b). Notably, PBMCs from PitNET patients clustered closer to tumor tissues compared with normal PBMCs (Fig. 1b), prompting a focused PCA comparing PitNET PBMCs against normal PBMCs. Among these samples, nine normal PBMCs were in-house generated (Supplementary Fig. 1g) and aligned well with publicly available normal PBMC profiles, reinforcing the distinct expression patterns observed in PitNET PBMCs (Fig. 1c and Supplementary Fig. 1h).

Differentially expressed genes (DEGs) between PitNET and normal PBMCs were identified using the limma^36^ package, incorporating gender and age as confounding covariates for adjustment. In total, 322 genes were significantly upregulated (log_2_|fold change | > 0.58, adjusted *P-*value < 0.05), and 685 were significantly downregulated (log_2_|fold change | < –0.58, adjusted *P-*value < 0.05) (Fig. 1d, Supplementary Table 2a). These differences were illustrated by a heatmap of DEGs (Supplementary Fig. 1i). Compared with normal PBMCs, Pathway enrichment analyses of the 322 upregulated genes in PitNET PBMCs pointed to pathways such as cytokine-cytokine receptor interaction and chemokine signaling (e.g., *CXCL5*, *CCL3L3*, *CXCL8*) (Fig. 1e, Supplementary Table 2b), consistent with enhanced cytokine activity in PitNET PBMCs. Gene set enrichment analysis (GSEA) confirmed a significant increase in the cytokine-cytokine receptor interaction pathway in PitNET PBMCs (Supplementary Fig. 1j). Additionally, PI3K-Akt signaling, cGMP-PKG signaling, and estrogen response emerged as enriched among upregulated genes (Fig. 1e). We also observed enrichment in pathways associated with circadian entrainment (e.g., *GNG11*, *GUCY1B1*) and hypertrophic cardiomyopathy (e.g., *ITGB3*, *ITGA2B*, *IL6*), suggesting systemic disruptions in PitNET PBMCs (Fig. 1e). In contrast, the 685 downregulated genes were associated with processes like inflammatory response, neutrophil extracellular trap formation, hematopoietic cell lineage, and natural killer cell-mediated cytotoxicity (Fig. 1f, Supplementary Table 2b), suggesting marked alterations in immune cell composition within PitNET PBMCs relative to normal PBMCs.

### Immune profiling of PitNET PBMCs and normal PBMCs reveals lymphoid and monocyte population imbalance

**Fig. 2:**
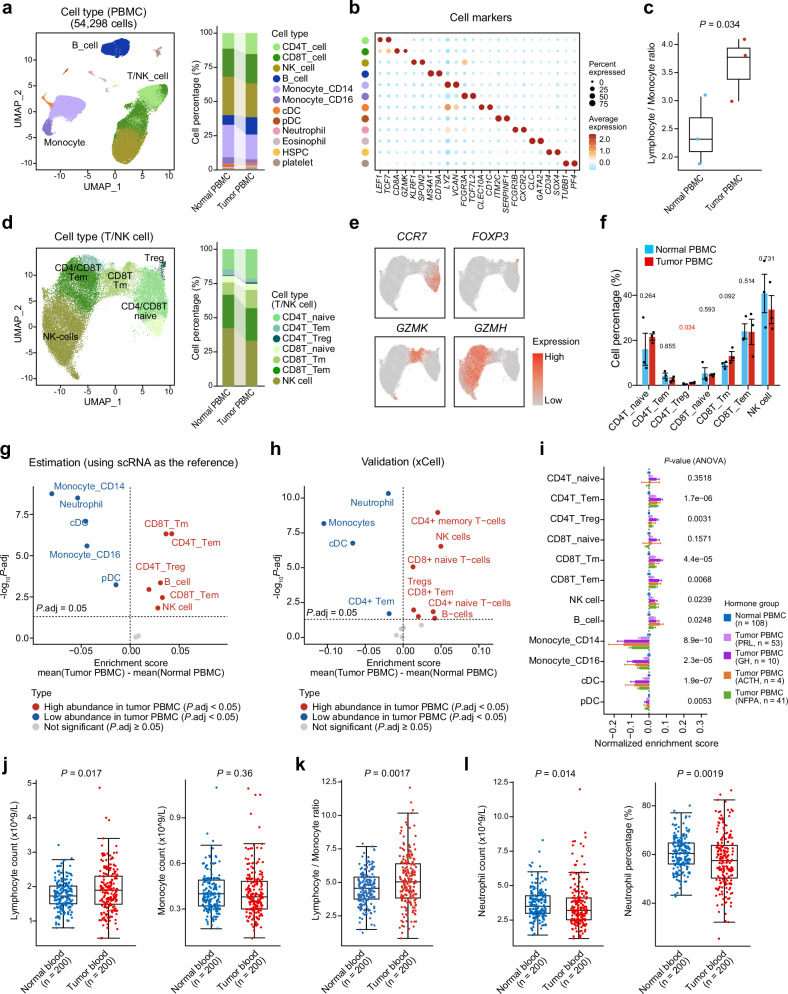
Immune profiling of PitNET PBMCs and normal PBMCs using scRNA-seq and estimation of bulk RNA-seq data. **a** Uniform Manifold Approximation and Projection (UMAP) plot of single-cell RNA-seq data for 3 PitNET PBMCs and 3 normal PBMCs (Total 54,298 cells). Cells are colored by annotated cell population (e.g., B cells, T/NK cells, monocytes). The stacked bar plot on the right quantifies the proportion of each cell type in PitNET PBMCs and normal PBMCs. cDC, conventional dendritic cells; pDC, plasmacytoid dendritic cells; HSPC, hematopoietic stem/progenitor cells; NK, natural killer cells. **b** Dotplot showing normalized expression (color scale) and percentage of cells expressing key marker genes (dot size) for each cell type. **c** Boxplot comparing the lymphocyte-to-monocyte ratio between PitNET PBMCs (*n* = 3) and normal PBMCs (*n* = 3). *P-*value was calculated using the Student’s *t test*. One-sided *P-*value was used. **d** UMAP plot illustrating the identification of T/NK cell subsets from PitNET PBMCs and normal PBMCs. **e** UMAP plot showing cells colored according to the expression of *CCR7*, *FOXP3*, *GZMK*, *GZMH*, respectively. **f** Barplot comparing the percentages of T/NK cell types between PitNET PBMCs (*n* = 3) and normal PBMCs (*n* = 3), with error bars representing the mean ± SE. *P-*values were calculated using the Student’s *t test*. One-sided *P-*values were used. **g** Cell type abundance estimation and comparison between PitNET PBMCs and normal PBMCs, using cell signatures generated from scRNA-seq data. *P-*values were calculated using the Mann-Whitney U test and adjusted using FDR. Two-sided *P-*values were used. **h** Cell type abundance estimation and comparison between PitNET PBMCs and normal PBMCs using the xCell^42^ algorithm. *P-*values were calculated using the Mann-Whitney U test and adjusted using FDR. Two-sided *P-*values were used. **i** Barplot comparing the estimated cell type abundance between PitNET PBMCs and normal PBMCs bulk RNA-seq, with error bars representing the mean ± SE. The estimated cell type abundance (presented in Supplementary Table 3) was normalized by subtracting the mean value of normal PBMCs. *P-*values were calculated using ANOVA. Two-sided *P-*values were used. **j** Boxplot comparing lymphocyte count (left panel) and monocyte count (right panel) from blood routine examination data between PitNET patients (*n* = 200) and healthy individuals (*n* = 200). *P-*values were calculated using the Mann–Whitney U test. Two-sided *P-*values were used. **k** Boxplot comparing lymphocyte-to-monocyte ratio between PitNET patients (*n* = 200) and healthy individuals (*n* = 200). *P-*value was calculated using the Mann–Whitney U test. Two-sided *P-*value was used. **l** Boxplot comparing neutrophil count (left panel) and neutrophil percentage (right panel) from blood routine examination data between PitNET patients (*n* = 200) and healthy individuals (*n* = 200). *P-*values were calculated using the Mann–Whitney U test. Two-sided *P-*values were used

To elucidate systemic immune alterations in PitNETs, we performed scRNA-seq on PBMCs from three treatment-naive PitNET patients and three healthy controls, yielding 54,298 high-quality cells after quality filtering. Batch effects were corrected using Harmony,^37^ enabling integrated visualization of PBMCs from both PitNET and normal donors (Supplementary Fig. 2a, b). Graph-based clustering with Seurat^38^ uncovered 17 distinct cell clusters (Supplementary Fig. 2c), which we annotated into 13 immune cell types based on canonical markers, for instance, T cells (*CD3E*), CD8^+^ T cells (*CD8A*), B cells (*CD79A*), monocytes (*CD14*), and others (Fig. 2a, b, Supplementary Fig. 2d). Notably, PitNET PBMCs exhibited a marked lymphoid-to-monocyte imbalance, characterized by increased proportions of T/NK cell and B cell alongside a reduction in monocytes (Fig. 2a). This skew also reflected by a significantly elevated lymphocyte-to-monocyte ratio in PitNET PBMCs relative to healthy controls (*P-*value = 0.034) (Fig. 2c). Further subclustering and annotation of T/NK cells revealed seven subsets (Fig. 2d, e, Supplementary 2e, f), including expansions of CD4^+^ regulatory T cells in PitNETs (CD4T_Treg; high *FOXP3* expression^39^, *P-*value = 0.034) (Fig. 2f).

To validate these findings at a larger scale, we applied single-sample Gene Set Enrichment Analysis (ssGSEA)^40,41^ to infer cell type abundances from bulk RNA-seq profiles of 108 PitNET PBMCs and 175 normal PBMCs, leveraging scRNA-seq-derived gene signatures of PBMCs (Fig. 2g, Supplementary Table 3a). The ssGSEA results showed a lymphoid bias in PitNET PBMCs, indicated by the enrichment of memory and effector-memory CD8^+^ T cells (CD8T_Tm and CD8T_Tem), effector-memory CD4^+^ T cells (CD4T_Tem), and regulatory CD4^+^ T cells (CD4T_Treg), alongside a reduction in monocyte subsets (monocyte_CD14 and monocyte_CD16) and a trend toward decreased neutrophil abundance (Fig. 2g, Supplementary Table 3b). These patterns were further corroborated by xCell^42^ (Fig. 2h). Additionally, relative to healthy controls and compared between different hormone groups, PitNETs with higher GH level exhibited the lower Monocyte_CD14 level but higher CD4T_Treg, CD4T_Tem and CD8T_Tm levels (Fig. 2i). Given potential correlations among hormone secretions in PitNETs (e.g., GH), endocrine activity may be related to peripheral immune changes (Supplementary Fig. 2g). Clinically, to validate these findings in an independent cohort, we performed a comprehensive blood routine examination on fresh blood samples from 200 PitNET patients and 200 age- and gender-matched healthy controls (Supplementary Table 4). This validation consistently mirrored the observations from scRNA-seq and bulk RNA-seq, revealing significantly elevated absolute lymphocyte counts (*P-*value = 0.017) (Fig. 2j, Supplementary Fig. 2h) and increased lymphocyte-to-monocyte ratios (*P-*value = 0.0017) (Fig. 2k) in PitNET patients compared to controls. Concurrently, neutrophil counts and percentages were significantly lower in the blood of PitNET patients, evidencing the trends observed in bulk PBMC analyses (*P-*value = 0.014 and 0.0019, respectively) (Fig. 2l).

### Postoperative immune reconstitution reveals dynamic lymphoid-myeloid rebalancing

**Fig. 3:**
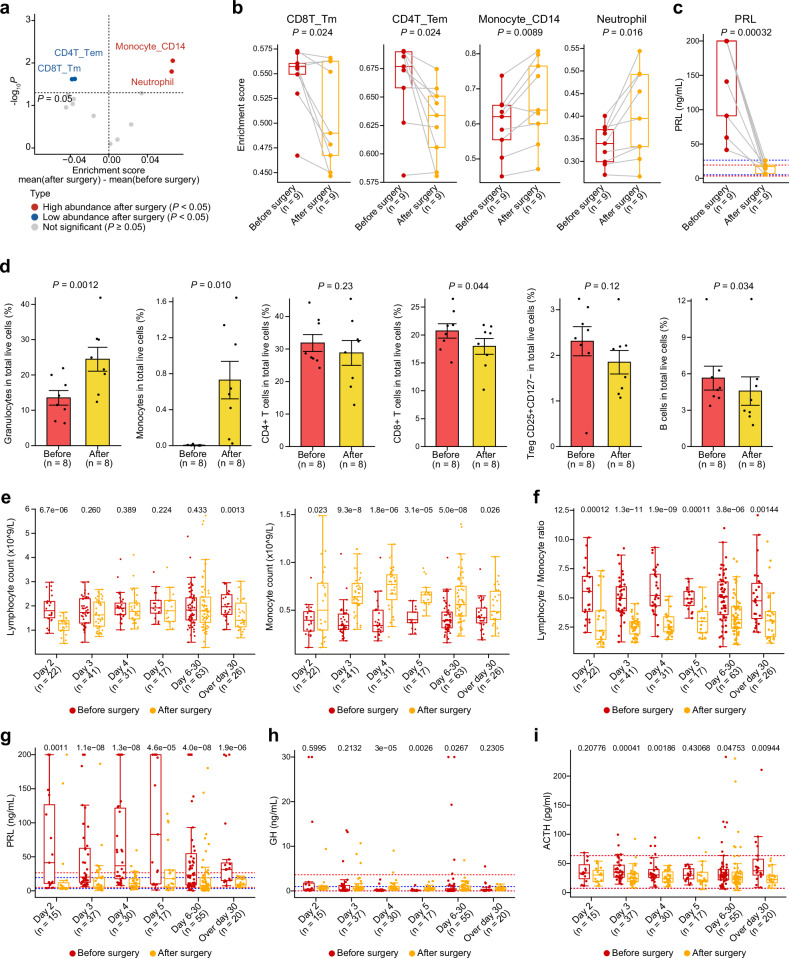
Postoperative peripheral immune recovery in PitNET patients. **a** Volcano plot illustrating changes in immune cell abundance scores (gene signatures for immune cell abundance estimation were derived from scRNA-seq) in paired PBMC samples from PitNET patients before and five days after surgery. Each dot represents an immune cell subtype, with blue indicating a decrease and red indicating an increase in abundance postoperatively. *P-*values were calculated using the paired Student’s *t test*. Two-sided *P-*values were used. **b** Boxplots comparing enrichment scores of four representative immune cell types (CD4T_Tem, CD8T_Tm, Monocyte_CD14 and neutrophil) before and five days after surgery. *P-*values were calculated using the paired Student’s *t test*. Two-sided *P-*values were used. **c** Boxplots comparing serum PRL levels before and five days after surgery. *P-*value was calculated using the paired Student’s *t test*. Two-sided *P-*values were used. The red dashed line indicates the normal reference range for females, and the blue dashed line indicates the normal reference range for males. **d** Barplots showing changes in the percentages of granulocytes, monocytes, CD4^+^ T cells, CD8^+^ T cells, Tregs (CD25^+^CD127^–^), and B cells among total live cells (*n* = 8) from PitNET patients before and five days after surgery. *P-*values were calculated using the paired Student’s *t test*. Two-sided *P-*values were used. **e** Boxplots depicting lymphocyte and monocyte counts from blood routine examinations of 200 PitNET patients (n in the figure refers to the number of samples that adds up to a total of 200), measured preoperatively and at various postoperative time points. *P-*values were calculated using the paired Mann-Whitney U test. Two-sided *P-*values were used. **f** Boxplots comparing the lymphocyte-to-monocyte ratio in the same cohort of 200 PitNET patients across preoperative and postoperative time points. *P-*values were calculated using the paired Mann–Whitney U test. Two-sided *P-*values were used. **g**, **h**, **i** Boxplots comparing serum PRL, GH, and ACTH levels in the same cohort (with available hormone records) across preoperative and postoperative time points. *P-*values were calculated using the paired Mann–Whitney U test (two-sided). The red dashed line indicates the normal reference range for females, and the blue dashed line indicates the normal reference range for males

Given the observed differences in peripheral immune profiles between PitNET patients and healthy individuals, we investigated whether removing the tumor would alleviate its systemic immunomodulatory influence. To achieve this, we analyzed matched preoperative (day 0) and five-day postoperative (day 5) PBMC samples from nine PitNET patients with markedly elevated PRL levels, and used bulk RNA-seq to infer immune cell abundances (Fig. 3a, Supplementary Table 5a). Notably, five days postoperative, several lymphocyte populations, including CD4T_Tem and CD8T_Tm, decreased markedly (*P-*value = 0.024), whereas monocyte levels (Monocyte_CD14) increased significantly (*P-*value = 0.0089) (Fig. 3b, Supplementary Table 5b). Consistently, PRL levels declined in the nine patients, returning to the normal physiological range (Fig. 3c, Supplementary Table 5c). To elucidate the molecular mechanisms underlying these shifts, we employed the limma package to DEGs between pre- and post-surgical samples. A total of 128 DEGs were identified, comprising 34 significantly downregulated and 94 significantly upregulated genes (Supplementary Fig. 3a). Among the upregulated genes, several myeloid-associated markers (e.g., *CD14*, *S100A12*, *S100A8*, and *S100A9*) were prominently increased (Supplementary Fig. 3a). Pathway enrichment analysis of the upregulated genes revealed enhanced activity in inflammatory response and neutrophil extracellular trap formation (Supplementary Fig. 3b). In contrast, the Hedgehog and Wnt signaling pathways were significantly downregulated (Supplementary Fig. 3c), suggesting broader implications for immune modulation.

To corroborate these transcriptomic findings, flow cytometry was performed on fresh PBMC samples from an additional eight patients collected before and five days after surgery (Supplementary Fig. 3d). These analyses confirmed significant postoperative increases in granulocytes (*P-*value = 0.0012) and monocytes (*P-*value = 0.010), along with notable reductions in CD8^+^ T cells (*P-*value = 0.044) and B cells (*P-*value = 0.034) (Fig. 3d). Further clinical validation was obtained from a larger independent cohort of 200 PitNET patients, using longitudinal blood routine examination data were collected preoperatively and at multiple postoperative intervals (from days 2 to 30 and beyond) (Supplementary Table 6). This analysis revealed a pronounced postoperative decrease in both lymphocyte count and percentage (most prominent between days 2 and 3), accompanied by a reciprocal increase in monocyte count and percentage peaking around days 3 to 5 (Fig. 3e, Supplementary Fig. 3e). Consequently, the lymphocyte-to-monocyte ratio decreased progressively (Fig. 3f, Supplementary Fig. 3f). Additionally, neutrophil count and percentage exhibited a transient yet substantial increase, particularly between postoperative days 2 and 5, further underscoring the expansion of the myeloid compartment (Supplementary Fig. 3g). In parallel, we observed declines in hormone levels (PRL, GH, ACTH) during postoperative days 3–5, with values returning to within normal ranges by over 30 days (Fig. 3g–i). Collectively, these results suggest that surgical resection of PitNETs may initiate a dynamic rebalancing of systemic immunity. The marked postoperative resurgence of myeloid cells, particularly monocytes and neutrophils, in conjunction with the contraction of specific lymphocyte subsets, highlights the critical impact of tumor-associated secretory dysregulation on systemic immune homeostasis and recovery.

### Lineage-specific differentiation and genomic instability shape PitNET secretory capacity

**Fig. 4:**
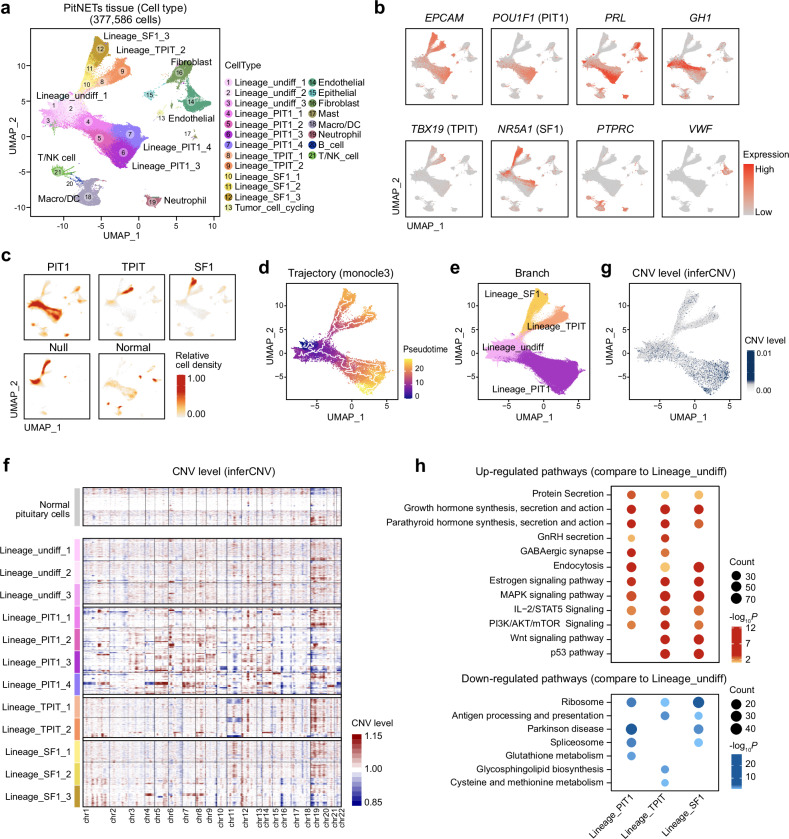
Single-cell analysis of PitNET tissues and identification of major cell types and CNV levels. **a** UMAP plot of 377,586 cells derived from PitNET tissues, colored by cell populations. These include tumor lineages (Lineage_undiff, Lineage_PIT1, Lineage_TPIT, Lineage_SF1), immune cells (T/NK cells, macrophages/dendritic cells, neutrophils), and stromal cells (endothelial, fibroblasts). **b** Feature plots displaying the expression patterns of key marker genes associated with specific tumor lineages, as well as immune and stromal cells, such as *EPCAM*, *POU1F1* (PIT1-lineage), *PRL* (PIT1-PRL-lineage), *GH1* (PIT1-GH-lineage), *TBX19* (TPIT-lineage), *NR5A1* (SF1-lineage), *PTPRC* (immune cells), and *VWF* (endothelial cells). Red indicates higher expression levels. **c** UMAP density plots highlighting the relative cell density for different PitNET lineages (PIT1-lineage, TPIT-lineage, SF1-lineage, Null cell lineage), as well as normal pituitary cells. Red corresponds to higher cell density. **d** Cell trajectory analysis using Monocle3, illustrating potential differentiation paths among various PitNET lineages. Cells are colored by pseudotime, transitioning from earlier states (purple) to more differentiated states (yellow). **e** UMAP plot of PitNET tissues, colored by cell branches. **f** Heatmap showing CNV levels across different PitNET tumor cell lineages as inferred using the inferCNV method. Each row represents a PitNET cell population, while the normal pituitary cells serve as the reference. CNVs are color-coded, with red indicating gain and blue indicating loss. **g** UMAP plot depicting inferred CNV levels across PitNET tumor cells, with higher CNV levels shown in darker blue. **h** Dotplot illustrating enriched pathways based on significantly upregulated (top) and downregulated (bottom) genes in PitNET cells compared to cells from Pre_branch. Dot size represents gene count, and color intensity represents statistical significance (-log_10_*P-*value). Two-sided *P-*values were used. Lineage_undiff: Lineage undifferentiation

Building on the observation of the peripheral immune impact, we next sought to elucidate the intrinsic cellular and molecular heterogeneity of PitNET tumor tissues. By analyzing single-cell sequencing data from 59 PitNET tumor tissues and 4 normal pituitary tissues, we constructed a comprehensive single-cell atlas of PitNETs (Supplementary Fig. 4a). After quality control, 377,586 cells were retained for subsequent analyses. Graph-based clustering identified 21 distinct cell populations, each defined by unique gene expression markers (Supplementary Fig. 4a, b). Notably, three major bifurcating clusters with high *EPCAM* expression, together with lineage-specific transcription factors *POU1F1*, *TBX19*, and *NR5A1*, were delineated as representing the three primary PitNET lineages (Fig. 4a, b). Tumor cells were organized into a common precursor population (Lineage_undiff_1 to Lineage_undiff_3) and three lineage-specific branches: the PIT1-lineage was subdivided into four subpopulations (Lineage_PIT1_1 to Lineage_PIT1_4), the TPIT lineage into two subpopulations (Lineage_TPIT_1 and Lineage_TPIT_2), and the SF1 lineage into three subpopulations (Lineage_SF1_1 to Lineage_SF1_3) (Fig. 4a, b). In addition, other cell groups included immune cells from the tumor tissue microenvironment (macrophages/dendritic cells, neutrophils, B cells, T/NK cells), stromal cells (epithelial, fibroblast, endothelial), and a minor population of cycling tumor cells (Fig. 4a and Supplementary Fig. 4b). We then categorized the cellular composition from PitNET patients and visualized it using density plots (Fig. 4c). The density plots revealed that cells within the PIT1-lineage PitNETs could be separated into two expression patterns: one subgroup exhibiting high *PRL* expression (PIT1-PRL subtype) and the other showing elevated *GH1* expression (PIT1-GH subtype) (Fig. 4b, c and Supplementary Fig. 4c). Additionally, cells from TPIT-lineage PitNETs were primarily localized in Lineage_TPIT_1 and Lineage_TPIT_2, while SF1-lineage cells were mainly distributed across Lineage_SF1_1, Lineage_SF1_2 and Lineage_SF1_3 (Fig. 4c and Supplementary Fig. 4c). Interestingly, non-hormone-secreting null cell PitNETs were primarily found in Lineage_undiff_1, Lineage_undiff_2 and Lineage_SF1_1, suggesting that terminal branch populations (e.g. Lineage_PIT1_3 and Lineage_PIT1_4) may exhibit heightened hormone secretion capabilities (Fig. 4c).

Using Monocle3,^43,44^ we reconstructed potential differentiation trajectories among PitNET tumor cell types (Fig. 4d). This analysis revealed that terminal branch cells likely originate from earlier intermediates (Lineage_undiff_1, Lineage_undiff_2, and Lineage_undiff_3), leading us to organize all cells into four overarching groups: Lineage_undiff, Lineage_PIT1, Lineage_TPIT, and Lineage_SF1 (Fig. 4e and Supplementary Fig. 4d, e). To characterize genomic alterations and assess copy number variation (CNV), we applied the inferCNV algorithm with normal pituitary cells as a reference. The results revealed that PitNET tumor cells harbor significantly elevated CNV levels compared to normal pituitary tissue, with terminally differentiated cells displaying greater CNV variability (Fig. 4f, g). For instance, within the PIT1-lineage, Lineage_PIT1_3 and Lineage_PIT1_4 demonstrated more pronounced CNV alterations relative to Lineage_undiff_1 (Fig. 4g). Subsequently, using Lineage_undiff as a reference, we identified DEGs in the terminal tumor cell branches and conducted enrichment analysis (Fig. 4g). Upregulated pathways were significantly associated with protein secretion as well as with growth hormone and parathyroid hormone synthesis, secretion, and action (Fig. 4h). In addition, key signaling pathways such as MAPK, IL-2/STAT5 and PI3K-AKT/mTOR were markedly upregulated in terminally differentiated cells (Fig. 4h). In contrast, downregulated pathways predominantly involved ribosome biogenesis (Fig. 4h). Overall, these findings illuminate the intrinsic heterogeneity of PitNET tumor cells and provide a detailed single-cell map of lineage-specific differentiation and CNV variability. Our atlas suggests that PitNETs are differentiation-associated tumors, with lineage-specific transcriptional programs and increasing genomic instability potentially contributing to their secretory specialization.

### Analysis of secreted proteins in PitNETs reveals lineage-specific secretion profiles

**Fig. 5:**
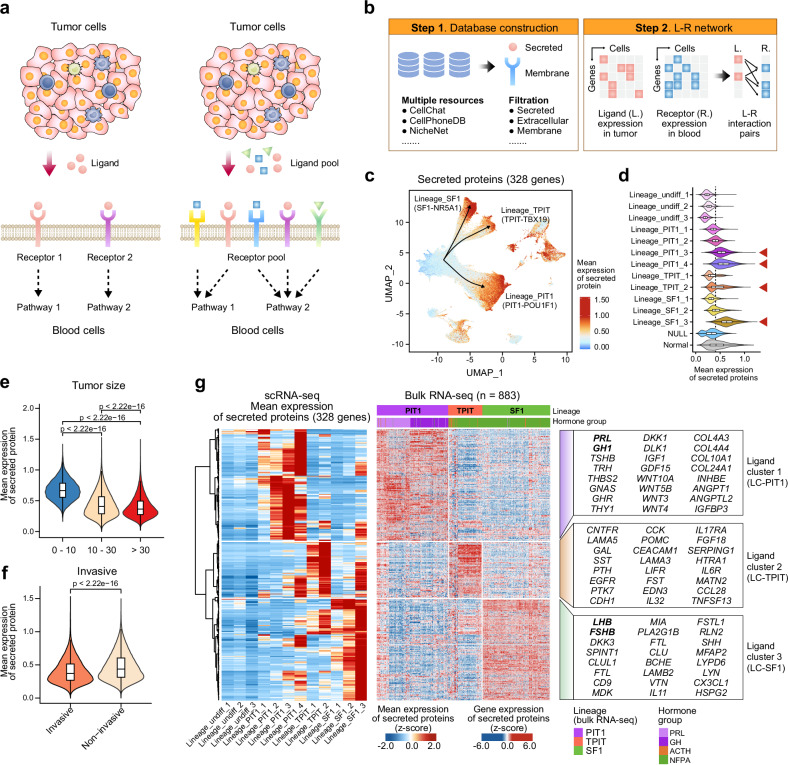
Lineage-specific secretion profiles in PitNETs. **a** Schematic illustrating cell-cell interactions driven by tumor-secreted proteins. PitNET cells release multiple ligands, which collectively form a ligand pool that binds to diverse receptor pools on blood cells, thereby activating various signaling pathways. **b** Workflow for constructing a comprehensive ligand-receptor (L-R) interaction network, integrating scRNA-seq data and curated interaction databases. **c** UMAP plot depicting the gene expression patterns of 328 secreted proteins across distinct PitNET lineages. Higher expression levels are shown in red, with prominent subpopulations labeled, such as Lineage_PIT1_3 (*PRL*), Lineage_PIT1_4 (*GH1*), Lineage_TPIT_2 (*TBX19*), Lineage_SF1_3 (*NR5A1*). **d** Violin plots comparing mean secreted protein expression across various PitNET lineages, highlighting significantly elevated secretion scores in terminally differentiated tumor cells (e.g., Lineage_PIT1_3, Lineage_PIT1_4) relative to earlier or intermediate cells (e.g., Lineage_undiff_1, Lineage_undiff_2). **e** Violin plot showing secreted protein expression in tumors of different sizes. *P-*values were calculated using the Mann–Whitney U test. Two-sided *P-*values were used. **f** Violin plot contrasting secreted protein expression between invasive and non-invasive PitNETs. *P-*values were calculated using the Mann–Whitney U test. Two-sided *P-*values were used. **g** Heatmap displaying mean expression of the 328 secreted proteins in scRNA-seq (left) and bulk RNA-seq (right) data. Clustering identifies three distinct ligand clusters corresponding to PIT1, TPIT, and SF1 lineages: Ligand Cluster 1 (LC-PIT1), Ligand Cluster 2 (LC-TPIT) and Ligand Cluster 3 (LC-SF1)

Next, we further explored the secretory characteristics of PitNET tumor cells. Secreted proteins, such as hormones and cytokines, are proteins encoded by specific protein‑coding genes, produced by cells and released into the extracellular environment via dedicated secretory pathways.^45^ In intercellular communication, many secreted proteins function as ligands that bind to receptors on target cells, thereby initiating signaling cascades that regulate processes like cell proliferation and differentiation.^46^ Since cells often secrete multiple proteins concurrently, cell-cell interactions typically involve a complex ligand pool (Fig. 5a). To capture this complexity, we compiled an extensive database of extracellularly detectable secreted proteins and integrated ligand-receptor interactions curated from 13 published cell-cell interaction tools, including CellPhoneDB,^46^ CellChat,^47^ NicheNet^48^ and others^49–54^ (Fig. 5b and Supplementary Fig. 5a). In all, our comprehensive database encompasses 1841 ligands, 1510 receptors, and 17,919 ligand-receptor interaction pairs (Supplementary Fig. 5b). Next, we identified 328 ligands that appear to be secreted proteins based on literature support, and that were also highly expressed in tumor cells (mean gene expression > 0.1 in scRNA-seq, variance >1 in bulk RNA-seq data) (Supplementary Fig. 5b and Supplementary Table 7). We then computed a secreted score based on the average gene expression levels of these proteins (Fig. 5c), and observed that terminally differentiated tumor cells, such as those in Lineage_PIT1_3 (*PRL*), Lineage_PIT1_4 (*GH1*), Lineage_TPIT_2, and Lineage_SF1_3, exhibited significantly higher secreted scores compared to earlier or intermediate cells (e.g., Lineage_undiff_1) (Fig. 5d).

Correlating secretion scores with clinical features revealed that tumors of smaller size, non-invasive characteristics, or hormone-staining positive status exhibited higher secretion capacity (Fig. 5e, f and Supplementary Fig. 5c). A heatmap of mean gene expression of these secreted proteins further confirmed that terminally differentiated tumor cells display elevated secretory activity (Fig. 5g). Unsupervised clustering of the gene expression of these secreted-proteins from scRNA-seq identified three distinct groups corresponding to the major PitNET lineages: ligand cluster 1 (LC-PIT1) for the PIT1 lineage, ligand cluster 2 (LC-TPIT) for the TPIT lineage, and ligand cluster 3 (LC-SF1) for the SF1 lineage (Fig. 5g). Bulk RNA-seq analysis of 883 PitNET tumor samples corroborated the association between secreted-proteins’ gene expression and PitNET subgroups, suggesting that these secreted proteins could serve as potential key markers for tumor classification (Supplementary Fig. 5d and Fig. 5g). For instance, LC-PIT1 encompasses genes related to hormone secretion (e.g., *PRL*, *GH1*, *TSHB*, *TRH*), MAPK signaling (e.g., *MET*), and WNT family (e.g., *WNT10A*, *WNT5B*, *WNT3*, *WNT4*) (Fig. 5g and Supplementary Fig. 5e). LC-TPIT primarily includes genes implicated in Cushing syndrome (e.g., *POMC*, *EGFR*), and LC-SF1 is enriched for components of the GnRH signaling pathway (e.g., *LHB*, *FSHB*) (Fig. 5g and Supplementary Fig. 5e).

### Tumor‑to‑blood ligand-receptor crosstalk and PBMC‑based classification of PitNET

**Fig. 6:**
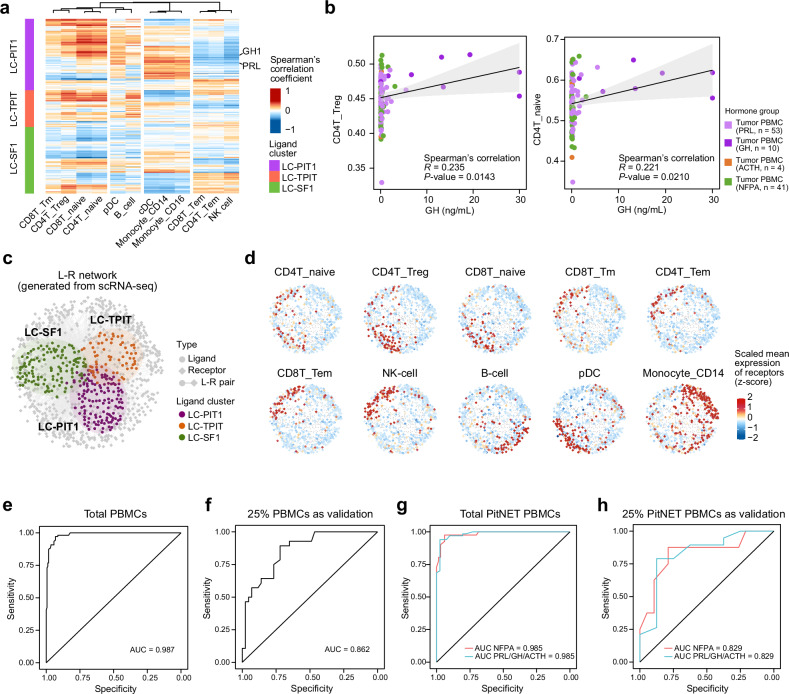
Tumor‑blood ligand-receptor crosstalk and PBMC‑based subtype prediction. **a** Heatmap of the Spearman’s correlation coefficients linking tumor‑ligand gene expression (bulk RNA‑seq) with estimated PBMC cell abundances in matched PitNET cases. Hierarchical clustering defines three immune modules. Red represents positive correlation and blue represents negative correlation. **b** Correlation between serum GH level and the estimated PBMC CD4T_Treg and CD4T_naive abundance levels. Each dot represents one patient. Spearman’s correlation coefficients and *P-*values were calculated. Two-sided *P-*values were used. **c** Ligand-receptor network constructed from scRNA-seq data. Three primary ligand clusters: LC-PIT1 (purple), LC-TPIT (orange), and LC-SF1 (green), are situated centrally, while receptors are distributed around the periphery based on their expression in PBMCs. **d** Expression levels of receptors within diverse immune cell populations (CD4T_naive, CD4T_Tem, CD4T_Treg, CD8T_naive, CD8T_Tm, CD8T_Tem, NKcell, B_cell, pDC, Monocyte_CD14) in the L-R network. The gene expression level was normalized by z-score. **e**, **f** ROC curves for the random forest model trained to classify PitNET patients from healthy individuals based on immune cell profiles in PBMCs. **e** Validation using all PitNET PBMC and normal PBMC samples. **f** Validation using 25% of PitNET PBMC and normal PBMC samples. **g**, **h** ROC curves for the random forest model trained to classify PitNET subtype (clinical NFPA, PRL/GH/ACTH) based on PBMC immune cell profiles. **g** Validation using all PitNET PBMC samples. **h** Validation using 25% of PitNET PBMC samples

To link tumor-derived secreted ligands with PBMC immune remodeling, we computed Spearman correlations between ligand expression in tumor tissues and cell abundances in PBMCs among the paired bulk RNA-seq samples (Fig. 6a, Supplementary Table 7a). PBMC cell populations clustered into four groups: naive/regulatory lymphoid group (CD4T_Treg, CD4T_naive, CD8T_naive, CD8T_Tm), B-cell related group (pDC, B cells), myeloid group (Monocyte_CD14, Monocyte_CD16, cDC), and effector-memory lymphoid group (CD8T_Tem, CD4T_Tem, NK cells) (Fig. 6a). In LC-PIT1, most ligands (including GH1) showed positive correlations with the naive/regulatory lymphoid group, particularly CD4T_Treg (Fig. 6a). Consistently, analysis of hormone secretion revealed that circulating GH levels were significantly associated with the abundance of CD4T_Treg, CD4T_naive, and CD8T_naive cells (Fig. 6b, Supplementary Fig. 6). This observation aligns with previous reports that GH supports CD4T_Treg function and maintenance while promoting the thymic output of naive CD4/CD8 T cells^55^ (Fig. 6b). To further validate these interactions, we analyzed PBMC scRNA-seq data filtered for receptor expression (mean expression > 0) and constructed a blood-based ligand-receptor network comprising 311 ligands, 662 receptors, and 3,334 ligand-receptor pairs (Fig. 6a, Supplementary Table 7b). The network topology, visualized using the Fruchterman-Reingold algorithm (R igraph package), placed ligands centrally and receptors peripherally (Fig. 6c). At the single-cell level, receptor expression displayed clear lineage specificity, with lymphoid subsets (CD4T_naive, CD4T_Tem, CD4T_Treg, CD8T_naive, CD8T_Tm, CD8T_Tem, NK cells, B cells) generally expressing fewer receptors than myeloid populations (pDC, Monocyte_CD14) (Fig. 6d), thereby demonstrating that ligands secreted by PitNETs can be mapped to corresponding receptor expression in peripheral blood.

Leveraging these differences, we trained a random‑forest classifier based on PBMC cell-type abundances to predict PitNET status. A total of 283 PBMC profiles (108 PitNET, 175 healthy) were randomly divided into training (75%) and validation (25%) sets. The model achieved 93.2% accuracy (AUC = 0.987) on the full dataset and 73.2% accuracy (AUC = 0.862) in the validation cohort (Fig. 6e, f). To further evaluate the translational potential, we trained a classifier to discriminate hormone-secreting (PRL/GH/ACTH) from non-functional (NFPA) PitNETs using 75% of the 108 PBMC samples. This model achieved an AUC of 0.985 in the full set and 0.829 in validation (Fig. 6g, h). Collectively, these results demonstrate that PBMC immune profiles not only distinguish PitNET patients from healthy individuals with high accuracy but also provide a practical framework for preoperative classification of functional versus non-functional subtypes, thereby offering potential utility in clinical decision-making and patient stratification.

### Estrogen-induced rat hyperprolactinemia recapitulates pituitary-immune endocrine axis dysfunction

**Fig. 7:**
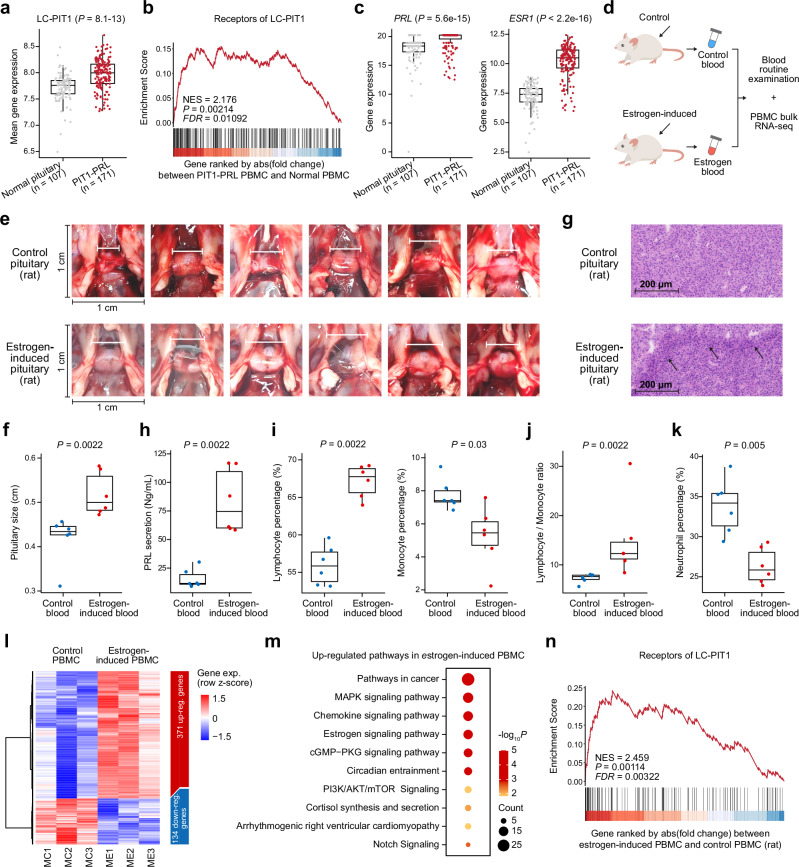
Estrogen-induced rat model recapitulates PitNET PIT1-PRL-lineage-like features and immune alterations. **a** Boxplot comparing mean gene expression levels of secreted proteins (LC-PIT1, ligand cluster 1 from Fig. 5g) in normal pituitary tissues (*n* = 107, GTEx dataset) and PIT1-PRL-lineage PitNET tissues (*n* = 171). *P-*value was calculated using the Mann–Whitney U test. Two-sided *P-*value was used. **b** GSEA plot illustrating receptor genes from LC-PIT1. Genes were ranked by the absolute fold change between PIT1-PRL-lineage PitNET PBMCs and normal PBMCs bulk RNA-seq. NES, normalized enrichment score. The *P-*value was calculated using GSEA. Two-sided *P-*value was used. **c** Boxplots depicting gene expression of *PRL* and *ESR1* in normal pituitary tissues (*n* = 107, GTEx dataset) compared to PIT1-PRL-lineage PitNET tissues (*n* = 171). *P-*values were calculated using the Mann–Whitney U test. Two-sided *P-*values were used. **d** Schematic outline of the estrogen injection protocol in male rats. Rats were injected with estrogen from weeks 1 to 6 (every other day), followed by no treatment during weeks 7 and 8. Peripheral blood and pituitary glands were collected at week 10 for further analysis. **e** Representative images of pituitary glands from control (upper row) and estrogen-induced rats (lower row). The estrogen-induced rats show visibly enlarged pituitary glands. **f** Boxplot comparing pituitary size (in cm) between control rats (*n* = 6) and estrogen-induced rats (*n* = 6). *P-*value was calculated using the Mann–Whitney U test. Two-sided *P-*value was used. **g** Histopathological examination of pituitary sections showing multi-hormonal nodules in rats. Sections were stained with hematoxylin and eosin (H&E). **h** Boxplot illustrating serum prolactin (PRL) levels in control versus estrogen-induced rats. *P-*value was calculated using the Mann–Whitney U test. Two-sided *P-*value was used. Box plot showing lymphocyte percentage and monocyte percentage (**i**), lymphocyte-to-monocyte ratio (**j**), and neutrophil percentage (**k**) in control and estrogen-induced rats. *P-*values were calculated using the Mann-Whitney U test. Two-sided *P-*values were used. **l** Heatmap of differentially expressed genes in PBMCs from control and estrogen-induced rats. Red indicates upregulated genes, while blue denotes downregulated genes. **m** The enriched pathways among upregulated genes in estrogen-induced rat PBMCs. Each dot represents a pathway calculated via Enrichr, colored by enrichment *P-*value and sized according to gene count. Two-sided *P-*values were used. **n** GSEA plot. The gene set comprises receptors from LC-PIT1. Genes were ranked by the absolute fold change between PBMCs of estrogen-induced rats and controls. The *P-*value was calculated using GSEA. Two-sided *P-*value was used

In PIT1-PRL-lineage PitNETs, the expression of LC-PIT1 ligands is significantly elevated relative to normal pituitary tissue (Fig. 7a), and simultaneously, the corresponding receptors in PBMCs display marked alterations compared to normal PBMCs (Fig. 7b). Given that estrogen modulates PRL secretion through feedback mechanisms within the hypothalamic-pituitary axis and considering the elevated expression of *PRL* and estrogen receptor 1 (*ESR1*) in PIT1-PRL-lineage PitNETs (Fig. 7c), we hypothesized that estrogen signaling might reproduce similar pathological and immune alterations. Based on previous reports,^56^ we established an estrogen-induced rat model to mimic the features of PIT1-PRL-lineage PitNETs. F344 rats received alternate-day estrogen from week 1 through week 6 (Fig. 7d). Treatment ceased for the subsequent two weeks (weeks 7–8) to reduce direct pharmacologic influence, and peripheral blood plus pituitary glands were collected in week 10. It revealed a significant increase in pituitary gland volumes in estrogen-treated rats compared to controls (6 estrogen-induced rats vs. 6 controls, *P-*value = 0.0022) (Fig. 7e, f). Histopathological examination (H&E) showed the presence of hyperplastic nodules composed of large eosinophilic cells in nearly all estrogen-induced pituitaries, consistent with prolactin-producing cell hyperplasia (Fig. 7g). Additionally, serum PRL levels were significantly elevated in estrogen-induced rats confirmed by ELISA (*P-*value = 0.0022) (Fig. 7h).

Consistent with clinical observations, estrogen-induced rats exhibited notable changes in peripheral immune cell populations, characterized by increased lymphocyte percentages (*P-*value = 0.0022, Fig. 7i), decreased monocyte percentages (*P-*value = 0.03, Fig. 7i), and consequently elevated lymphocyte-to-monocyte ratio (*P-*value = 0.0022, Fig. 7j). Neutrophil levels were notably reduced (Fig. 7k). Analysis of cell-type abundance in PRL-PitNET patients revealed a tendency toward increased lymphocyte populations, specifically CD4T_Tem, CD4T_Treg, and CD8T_Tm cells (Supplementary Fig. 7a). Then, peripheral blood from every two rats was pooled, and bulk RNA-seq was performed on the isolated PBMC. Differential expression analysis between estrogen-induced rats and controls identified 371 significantly upregulated and 134 significantly downregulated genes (Supplementary Fig. 7b and Fig. 7l, Supplementary Table 8a). Enrichment analysis of upregulated genes highlighted pathways including pathways in cancer, MAPK, chemokine, and estrogen signaling (Fig. 7m, Supplementary Table 8b). Furthermore, GSEA revealed significant enrichment of receptors corresponding to the LC-PIT1 ligand cluster in estrogen-induced rat PBMCs relative to controls (Fig. 7n). These findings suggest that estrogen-driven feedback mechanisms within the hormone-pituitary-immune axis may influence systemic immune regulation.

### Discussion

The pituitary gland orchestrates systemic homeostasis through a finely regulated secretome-peripheral axis, yet in PitNETs, pathological hypersecretion disrupts this balance, driving a cycle of hormonal excess and immune dysregulation. Our integrative analysis of 1976 PitNET and control samples redefines PitNETs as systemic modulators of peripheral immunity rather than isolated endocrine lesions. We identified distinct molecular subgroups and hormone groups aligned with major pituitary lineages and uncovered a lymphocyte-dominant immune imbalance in patients, corroborated by bulk and single-cell transcriptomics. Notably, serum GH levels were positively correlated with the abundance of naive/regulatory T-cell populations, consistent with previous reports and underscoring the immunomodulatory role of pituitary hormones.^57^ Previous studies also demonstrated that GH promotes lymphoid cell proliferation, enhances thymopoiesis to increase naive T-cell output, and supports cell survival through anti-apoptotic signaling pathways such as PI3K/Akt and NF-κB.^58,59^ PitNETs often exhibit multi-hormonal secretion, further contributing to the complexity of peripheral immune states. Postoperative profiling indicated that surgical debulking can partially restore immune balance, highlighting the lymphocyte-to-monocyte ratio as a potential biomarker for monitoring therapeutic response.

The study demonstrates a marked lymphoid-to-monocyte imbalance in PBMCs from PitNET patients, with increased lymphocyte counts and decreased monocyte and neutrophil levels. These alterations are indicative of systemic immune dysregulation driven by tumor-secreted factors. The lineage-specific secretory profiles identified in PitNETs reveal a complex network of ligand-receptor interactions that likely contribute to this imbalance. For instance, the LC-PIT1 secretome exhibits immunomodulatory properties that enhance T cell recruitment.^60^ This aligns with prior reports linking prolactin hypersecretion to lymphocyte proliferation and altered myeloid differentiation. Such skewed immune dynamics may transiently bolster adaptive immunity but ultimately compromise innate surveillance, creating a permissive niche for tumor progression. Beyond these systemic effects, accumulating evidence indicates that immune infiltrates within PitNETs also have prognostic significance. In particular, CD8^+^ T lymphocyte density has been inversely associated with cavernous sinus invasion and resistance to first-generation somatostatin analogs in somatotroph tumors, while CD68^+^ macrophage infiltration correlates with tumor size, invasiveness, and treatment response.^61,62^ Our work expands these observations that immune signatures may serve as biomarkers to predict tumor behavior and therapeutic outcomes, and their longitudinal assessment could complement hormonal monitoring during follow-up.

While our multi-omics approach provides unprecedented resolution of PitNET-immune interactions, several limitations warrant consideration. First, scRNA-seq PBMC profiling was conducted on a small number of patients, which may limit the generalizability of findings regarding peripheral immune heterogeneity. Second, the number of ACTH-lineage tumor samples was also limited. Third, transcriptomic data alone cannot fully capture post-translational modifications or the bioavailability of secreted proteins. Thus, integration with proteomic and metabolomic datasets will be essential in future studies. Fourth, longitudinal monitoring beyond one year post-surgery is needed to assess long-term immune recovery and its relationship with tumor recurrence. Fifth, although trajectory analysis identified a putatively undifferentiated lineage cell population, canonical pituitary progenitor markers (e.g., SOX2, SOX9) were expressed at low levels, leaving the cellular origin of PitNETs unresolved. Finally, while CD4⁺ and CD8⁺ effector-memory populations were enriched in both tumor and peripheral compartments, their functional impact on tumor immunity and systemic surveillance remains to be fully elucidated, underscoring the need to dissect tumor-peripheral immune crosstalk.

Following tumor removal, surgical resection of PitNETs initiates dynamic immune rebalancing, characterized by myeloid resurgence (monocytes/neutrophils) and lymphoid contraction, a phenomenon validated across transcriptomic, flow cytometry, and clinical cohorts. Short-term immune shifts from surgical stress are typically acute and transient, peaking within the first postoperative day and normalizing within 2–3 days. For example, neurosurgical patients exhibit a ~ 70% increase in circulating monocytes within hours, which returns to baseline by 72 h,^63^ with similar trends observed in colorectal surgery.^64^ Given the minimally invasive transsphenoidal approaches in our cohort, the persistent immune alterations at postoperative day 5 likely reflect the immunological consequences of PitNET removal and restoration of endocrine homeostasis rather than surgical trauma alone. Additionally, while perioperative glucocorticoid administration in some patients may transiently influence immune and endocrine states, it was discontinued by postoperative day 3, and blood sampling on day 5 was therefore expected to capture endogenous recovery. The transient postoperative neutrophil surge likely reflects acute inflammation resolution, while monocyte recovery suggests restored homeostatic signaling upon tumor removal. Critically, these findings position the lymphocyte-to-monocyte ratio as a clinically actionable biomarker for monitoring therapeutic efficacy and recurrence risk. Furthermore, SF1-lineage tumors demonstrate distinctly different recurrence patterns based on the extent of surgical resection. Complete resection is associated with recurrence rates below 5%, whereas subtotal resection yields significantly higher recurrence rates ranging from 15 to 35%.^65^ In this context, our novel blood-based immunological biomarker panel may serve as an early indicator of tumor recurrence, thereby providing crucial guidance for determining the necessity and timing of adjuvant medical therapy or radiation treatment. Meanwhile, the estrogen-induced rat model recapitulated key features of PIT1-PRL lineage tumors, including prolactin hypersecretion and peripheral immune skewing. Sustained estrogen exposure drove an ESR1-PRL autocrine loop, mirroring clinical observations of hormone receptor upregulation in aggressive PitNETs.^66,67^ These parallels suggest that the model may help explore how tumor-immune interactions contribute to disease and could provide a platform for testing targeted therapies aimed at restoring immune balance.

In summary, our multi-omics analyses establish PitNETs as endocrine drivers of systemic immune remodeling. By integrating transcriptomic and single-cell data, we have delineated the intrinsic secretory activity of PitNETs and their impact on peripheral immune dysfunction, characterized by a lymphocyte-monocyte imbalance. This detailed map of tumor heterogeneity and immune interactions offers promising avenues for therapeutic intervention. Future research should aim to elucidate the precise mechanisms by which PitNETs influence immune cell function and develop targeted therapies to disrupt these interactions, advancing our understanding of PitNET biology and its systemic effects on the immune system.

## Materials and methods

### Patients

Patient specimens and blood samples were collected with the approval and consent of the hospital’s ethics committee. Patients were recruited from two Chinese cohorts, comprising a total of 1178 individuals diagnosed with pituitary neuroendocrine tumors (PitNETs) who underwent surgical resection at Ruijin Hospital (the “Ruijin cohort”) or Beijing Tiantan Hospital (the “Tiantan cohort”) (Supplementary Table 1). The sequencing data and clinical information of 341 PitNET patients (317 bulk RNA-seq and 24 scRNA-seq) from the Tiantan cohort were obtained from previously published work.^3,15^ In the Ruijin cohort, all PitNET patients were enrolled between January 2017 and December 2024, and comprehensive transcriptomic profiling was performed using bulk RNA-seq, scRNA-seq of tumor tissues, as well as bulk RNA-seq and scRNA-seq of PBMCs. An independent validation cohort of 208 PitNET patients was collected from January to December 2024; this cohort included 8 patients assessed by flow cytometry and 200 patients who underwent routine blood examinations before and after surgery. An additional 200 healthy controls for blood examinations were randomly selected from the contemporaneous Ruijin Hospital Physical Examination Center, with their age and sex matching the distribution of the PitNET cohort. Hormone measurements were not available for the 200 healthy controls; instead, established normal ranges were used to assess hormone status. All PitNET patients in the Ruijin cohort received surgical treatment at the Department of Neurosurgery at Ruijin Hospital, Shanghai Jiao Tong University School of Medicine, and their clinical data were retrospectively obtained from medical records. For PitNET patients in the Ruijin cohort, additional clinical features were incorporated, including visual defect status, hormone staining (positive/negative), medical history (with or without hormone therapy), and lineage-specific transcription factor staining (TPIT, PIT1, SF1). For the PBMC and routine blood cohorts, detailed hormonal data were collected. Detailed sample information and clinical characteristics were listed in Supplementary Table 1.

### Sample collection

For PitNET tissue dissociation and primary tumor cell extraction, tissues were transported in tissue storage solution (Miltenyi Biotec, Cat. no. 130-100-008) on ice to maintain viability. Tissues were washed 2–3 times with phosphate-buffered saline (PBS; Hyclone, Cat. no. SH30256.01) before being minced on ice. A dissociation enzyme cocktail, comprising 1 mg/ml Type VIII Collagenase (Sigma-Aldrich, Cat. no. C2139), 2 mg/ml Dispase II (Sigma-Aldrich, Cat. no. 4942078001), 1 mg/ml Trypsin Inhibitor (Sigma-Aldrich, Cat. no. T6522), and 1 unit/ml DNase I (NEB, Cat. no. M0303S) dissolved in serum-free DMEM, was used to digest the tissues. The neoplastic tissues were incubated at 37 °C with shaking at 50 r.p.m. for approximately 40 min, and dissociated cells were collected at 20-min intervals to maximize yield and viability. The resulting cell suspensions were filtered through a 40 μm nylon cell strainer (Falcon, Cat. no. 352340), and red blood cells were removed using RBC lysis buffer (Invitrogen, Cat. no. 1966634) with 1 unit/ml DNase I. The cells were then washed with PBS containing 0.04% bovine serum albumin (BSA; Sigma-Aldrich, Cat. no. B2064) using a stepwise decreasing centrifugation speed with increased duration. Finally, cell viability was assessed by staining with 0.4% Trypan blue (Invitrogen, Cat. no. T10282), and the cells were cultured in DMEM supplemented with 10% FBS and 1% antibiotic mixture.

Peripheral blood samples were collected preoperatively and postoperatively to assess systemic immune profiles. Preoperative samples were obtained in the morning (6–8 a.m.) upon hospital admission, typically about 24 h before surgery. At this stage, patients had not received glucocorticoid replacement, stress-dose coverage, or any other hormone therapy. Perioperative hydrocortisone was administered intraoperatively and for the first 24–48 h post-surgery as stress-dose coverage, with routine discontinuation on day 3 unless adrenal insufficiency was confirmed. Given the short elimination half-life of hydrocortisone (t½ ≈ 1.5–2 h), its circulating concentrations are expected to decline rapidly after cessation. By postoperative day 5, any exogenous hydrocortisone levels would have been negligible and well below the threshold for significant immunomodulatory effects. No patients received supraphysiologic stress doses, minimizing confounding effects of exogenous glucocorticoids. All sample collection and processing procedures were approved by the Ethics Committee of Ruijin Hospital, under approval IDs 2019-39 and 2023-209.

PBMCs were isolated using lymphocyte separation medium (40504ES60, YEASEN, China) according to manufacturer’s instruction. 1 × 10^6 cells were used to perform flow cytometry or bulk RNA-seq. To extract PBMCs, we first collected fresh blood in an EDTA-treated anticoagulant tube and then diluted the blood sample with an equal volume of phosphate-buffered saline (PBS) in a 15 mL conical tube. Diluted blood was carefully layered over an equal volume of Ficoll-Paque solution in a new 15 mL conical tube by gently pipetting the blood along the tube side to avoid mixing. The tube was centrifuged at 400 x *g* for 30 min at room temperature without braking to separate the layers. After centrifugation, the white and cloudy PBMC layer which is located between the plasma (top layer) and the Ficoll-Paque (bottom layer) was collected and transferred to a new 15 mL conical tube. The PBMCs were then washed with PBS and centrifuged at 300 × *g* for 10 min. The washing step was repeated twice to remove any remaining platelets and Ficoll. Finally, PBMC pellet was resuspended in a PBS buffer with 1% BSA for flow cytometry or bulk RNA-seq.

### Bulk RNA sequencing of PitNET tissues and PBMCs

RNA library preparation was performed using the Kapa RNA HyperPrep Kit with RiboErase (Roche, Switzerland). A total of 500 ng RNA was fragmented to an average size of 200–300 bp at 94 °C for 6 min. The cleaved RNA fragments were reverse transcribed into first-strand cDNA using reverse transcriptase and random primers, followed by the synthesis of the second-strand to form double-stranded cDNA. After A-tailing, adapter ligation, and PCR enrichment, the final library was quantified using 4200TapeStation (Agilent Technologies, USA). Indexed RNA-Seq libraries were sequenced at the MGISEQ 2000 platform (MGI + China) according to a PE 150 bp protocol in the National Research Center for Translational Medicine (Shanghai).

### Bulk RNA sequencing alignment

To quantify expression profiling in PitNET tissues, PBMCs, and normal controls, raw sequencing reads were mapped to the Genome Reference Consortium Human Build 38 (GRCh38, Release 45). Both the genome and annotation files were downloaded from the GENCODE database (https://www.gencodegenes.org/human/). Alignment was performed using STAR (v2.7.11a).^68^ RSEM (v1.3.1)^69^ was then used to generate read counts, as well as TPM (transcripts per kilobase of exon per million mapped reads) and FPKM (fragments per kilobase of transcript per million mapped reads) metrics for all PitNETs tissue and PBMC samples. For quantification purposes, relative gene expression was measured by applying a variance stabilizing transformation (VST) with the R package DESeq2.^70^ Principal component analysis (PCA) was conducted using the R package stats. Differentially expressed genes (DEGs) between different conditions were subsequently calculated using the R package limma.^36^ The significance level to define DEGs in bulk RNA-seq was set as adjusted *P-*value < 0.05 and log2 fold change > 0.58 (fold change 1.5) for up-regulation and adjusted *P-*value < 0.05 and log2 fold change < –0.58 for downregulation.

### Single-cell RNA sequencing alignment and generation of gene expression matrix

Raw sequencing reads from the cDNA library were processed using the BD Rhapsody Whole Transcriptome Assay Analysis Pipeline (v1.8). This pipeline performed quality filtering of raw reads, annotated reads and unique molecular identifiers (UMIs), identified putative cells, and generated a comprehensive single-cell expression matrix. In addition, the pipeline used the SampleTag library and its Sample Determination algorithm to assign each cell to its originating sample with high accuracy. From the pipeline outputs, the UMI count matrix, with gene expression details at each individual cell level, was selected for downstream analysis. Reads were aligned to the human reference genome (GRCh38) to ensure precise mapping. Together, these procedures established a robust framework for subsequent single-cell transcriptomic analysis.

### Quality control of single-cell expression profile data

Gene expression matrices were imported into R and analyzed using the Seurat^38^ package. For single-cell quality control, in the initial step, we conducted a filtration process to eliminate cells exhibiting low gene expression. Cells with expressed genes < 200, or unique counts >50,000 or <500, or expressed mitochondrial RNA > 30% were removed for quality control. Doublets were identified and removed using the DropletUtils (https://bioconductor.org/packages/DropletUtils/) package. Upon analyzing the cell distributions across the 69 samples, to ensure effective integration, we employed random downsampling to limit the maximum number of enrolled cells for each sample to 10,000 using the R sample function. Ultimately, a total of 377,586 cells from PitNET tumor and normal pituitary tissues, and 54,298 cells from tumor and normal PBMCs were included for downstream data analysis. Following the generation of the Seurat object, we calculated the top 2000 highly variable genes using the selection method “vst” and utilized them to conduct principal component analysis (PCA). Batch effects were adjusted across samples using the Harmony^37^ R package, with ‘max_iter’ set to 3. Subsequently, the top 20 harmony coordinates were chosen for clustering analysis and dimensionality reduction. Dimensionality reduction method Uniform Manifold Approximation and Projection (UMAP)^71^ was performed for visualization. The graph-based unsupervised clustering was used to identify cell clusters with the resolution set to 0.8 in the “FindClusters” function.

### Cell type annotation of scRNA-seq data

After unsupervised clustering, marker genes for each cluster were calculated using the “FindAllMarkers” function and defined under the following criteria: log2(fold changes) (log2FC) > 0.58 (FC > 1.5), min.pct >0.1, and adjusted *P-*value < 0.05. Cells were annotated using both machine-learning-based software SingleR^72^ and high expression of canonical markers (i.e., *EPCAM* for epithelial cells, *NCAM1* for neuron cells, *VWF* for endothelial cells, *DCN* for fibroblast, *PTPRC* for immune cells, *C1Q* for macrophage/dendritic-cells, and *CD3* for T cells, *CD79A* for B cells). The tumor cell identification was described as (i) expression of neuron markers (*NCAM1*) and canonical markers of PitNET tumor tissues, such as *POU1F1*, *TBX19*, and *NR5A1*; (ii) higher copy number variation level. The copy number karyotyping analysis was performed by inferCNV (https://github.com/broadinstitute/infercnv) with default parameters. Cells from normal pituitaries were used as the reference for copy number variation (CNV) analysis.

### Differentially expressed genes calculation in scRNA-seq

We employed Seurat’s “FindMarkers” function to detect genes expressed at varying levels between distinct cell types. The log fold change (logFC) threshold was set to 0.01, and the minimum percentage of cells expressing the gene (min.pct) was set to 0.01. DEGs were defined as those with log2FC > 0.58 & adjusted *P-*value < 0.05.

### Generation of cell signatures based on scRNA-seq

The top 200 expressed genes (log2FC > 0.58 & adjusted *P-*value < 0.05) in each cell type were selected as an in-house cell marker database of PitNETs tissues and PBMCs, which was enrolled for cell type abundance estimation analysis on bulk RNA-seq data. The enrichment score to infer cell abundance from bulk RNA-seq was calculated using a single-sample gene set enrichment analysis (ssGSEA) algorithm via the R GSVA^41^ package.

### Trajectory analysis of tumor cells

To assess dynamic changes in cell state across PitNET tumor subclusters, we performed trajectory analysis using Monocle3 (version 1.3.7).^73^ We used UMAP embeddings to order cells in pseudotime along the inferred trajectory and cells in Pre_branch_1 were defined as the initiating point of the trajectory.

### Ligand-receptor network analysis

The ligand-receptor (L-R) network database was assembled by integrating 17,919 L-R pairs, (comprising 1841 ligands and 1510 receptors) collected from 13 public databases, including NicheNet (version 2.1.5),^48^ CellPhoneDB (version 5.0.0),^46^ CellChatDB (version 1.6.1),^47^ CCIDB_Human (version 202408),^53^ CellCall (version 1.0.7),^51^ CellCellInteractions (version 202311, https://baderlab.org/CellCellInteractions), Cellinker (version 202408),^49^ CellTalkDB (version 202011),^74^ CITEdb (version 1.0),^54^ iTALK (version 0.1.0, https://github.com/Coolgenome/iTALK), LIANA (version 0.1.14),^50^ Ramilowski etal. (version 2015),^75^ and scConnect (version 1.0.4, https://github.com/JonETJakobsson/scConnect). Ligands exhibiting high tumor expression (mean>0.1 in scRNA‑seq and variance>1 in bulk RNA‑seq) were then filtered, resulting in 328 candidate secreted factors. Receptors expressed in PBMCs (mean>0 in scRNA‑seq) were similarly screened to retain 967 receptors. These subsets were intersected with the master compendium to yield a refined network of 311 ligands, 662 receptors, and 3,334 L-R pairs. The secreted score was calculated for each lineage cluster by averaging the scaled expression of its ligands in tumor cells. The network was visualized using R igraph with the Fruchterman-Reingold layout, positioning ligands centrally and receptors peripherally to illustrate how PIT1, TPIT, and SF1 lineage secretomes engage cognate receptors on peripheral immune cells.

### Functional enrichment analysis

For functional enrichment, we performed Gene Set Enrichment Analysis (GSEA)^76^ using the pre‑ranked algorithm in conjunction with the R package clusterProfiler.^77^ Gene sets were obtained from the Molecular Signatures Database (MSigDB, v2023.1),^78^ including the HALLMARK collection of 50 curated biological processes and the Kyoto Encyclopedia of Genes and Genomes (KEGG)^79,80^ pathway database. A significance threshold of *P-*value < 0.05 was applied throughout, and enrichment results were visualized using the enrichplot R package (https://bioconductor.org/packages/enrichplot/). In addition, we conducted independent enrichment analysis on differentially expressed genes using the Enrichr web service (https://maayanlab.cloud/Enrichr/),^81^ applying the KEGG_2021_Human and MSigDB_Hallmark_2020 libraries (https://maayanlab.cloud/Enrichr/#libraries). Terms with *P-*values < 0.05 were considered significant.

### Flow cytometry

For surface staining, cells were resuspended in 50 μl PBS containing antibody cocktails and stained at room temperature in the dark for 30 min. For intracellular staining, fresh PBMC cells were fixed and permeabilized by Foxp3 Fixation/Permeabilization kit (Thermo Fisher Scientific) at 4 °C for 45 min and stained with 50 μl of 1× permeabilization buffer containing antibody cocktails at 4 °C in the dark for 45 min. The gating strategy for immunophenotyping of human pituitary tumor cell suspensions was described below. Briefly, CD45-positive cells were divided into T cells (CD45^+^CD3^+^), B cells (CD45^+^CD3^-^CD19^+^), NK cells (CD45^+^CD3^-^CD19^-^CD56^+^). All flow data were acquired by BD FACSDiva software v8.0.2 and analyzed by FlowJo VX.

### Blood routine examination

To perform blood routine examination, peripheral blood was collected from rat at regular intervals. Blood samples treated with heparin anticoagulation were used for analyzing their cellular component by Automated Hematology Analyzer XN series (XN-10[B4]).

### Calculation of the lymphocyte-to-monocyte ratio

For scRNA-seq data, the lymphocyte-to-monocyte ratio was calculated by (sum of lymphocyte cell percentages) / (sum of monocyte cell percentages). The lymphocyte cells include B_cell, CD4T_cell, CD8T_cell, and NK_cell, and the monocyte cells include Monocyte_CD14, Monocyte_CD16. For blood routine examination data, the lymphocyte-to-monocyte ratio was calculated by (lymphocyte cell percentage) / (monocyte cell percentage).

### Rat prolactinoma model

In the rat prolactinoma model, twenty 4-week-old male F344 rats were purchased from Vital River Laboratories (Beijing, China) and maintained in a controlled 12-h light/12-h dark environment with ad libitum feeding. The local animal care and use committee approved all experimental protocols. Prolactinomas were induced by administering 17β-estradiol for 6 weeks, as described by Cao et al. and Lin et al.^66,82^ Following the induction period, blood was collected from the inner canthus of the rats to measure serum prolactin (PRL) levels using ELISA and to perform a complete blood count. After euthanasia, pituitary tissues were collected for hematoxylin and eosin (H&E) staining and immunohistochemistry, and additional blood samples were harvested and sent for bulk RNA sequencing. The rat experiments in this study were approved by the Ruijin Hospital Ethics Committee, with the approval by Institutional Animal Care and Use Committee (IACUC: B-2021-004).

### Tissue histology and immunostaining

For H&E staining, tissues were fixed in 4% paraformaldehyde overnight at room temperature (RT), followed by dehydration in 70% ethanol. Tissues were embedded in paraffin, sectioned at a thickness of 5 μm, and stained with H&E following the standard protocol.

### ELISA assay

Blood was collected from rats, allowed to clot, and centrifuged to obtain serum; PRL concentrations were then measured using an ELISA kit (Elabscience, E-EL-R3006).

### Statistical analysis

For statistical analysis: (I) Two-group comparisons: medians were compared using the Mann–Whitney U test. For very small samples with fewer than six units per group, means were compared using a Student’s *t test* for unpaired data. (II) Comparisons involving multiple groups: analysis of variance (ANOVA) was used to compare mean values when more than 2 groups were compared. (III) Adjustment of *P-*values: we applied the Benjamini-Hochberg (BH, alias FDR) method to adjust *P-*values. In the calculations related to differentially expressed genes and cell type abundance scores, we employed adjusted *P-*values to control the false positive rate, with a threshold of adjusted *P-*value < 0.05. Regarding GSEA, significance was established at *P-*value < 0.05, with an additional adjustment threshold of adjusted *P-*value < 0.25. For other statistical analyses, significance was determined at the conventional threshold of *P-*value < 0.05. All statistical analyses were conducted using R (https://www.r-project.org/).

## Supplementary information

Supplementary Materials - Supplementary Fig. 1 to 7

Supplementary Table 1

Supplementary Table 2

Supplementary Table 3

Supplementary Table 4

Supplementary Table 5

Supplementary Table 6

Supplementary Table 7

Supplementary Table 8

## Data Availability

Raw sequencing data generated in this study have been deposited at the National Omics Data Encyclopedia (NODE) with the accession number OEP00006242. The processed bulk RNA-seq and scRNA-seq data are accessible on NODE (OEZ00021642 and OEZ00021641 for bulk RNA-seq, OEZ00021643 and OEZ00021640 for scRNA-seq). This paper also analyzes existing and publicly available data, which are listed in Supplementary Table 1a–c.

## Code availability

The code used for the data analysis in this paper is available on GitHub: https://nrctm-bioinfo.github.io/PitNETs_project_2025.
