# Supplementary material for: Multidimensional tumor-blood profiling uncovers systemic lymphocyte-monocyte imbalance in pituitary neuroendocrine tumors
Source: Signal Transduct Target Ther. 2025 Nov 18;10:377. doi: 10.1038/s41392-025-02489-0 (PMC12627556; doi:10.1038/s41392-025-02489-0)
Supplement: Supplementary file 1 — Supplementary Materials - Supplementary Fig. 1 to 7 [file 41392_2025_2489_MOESM1_ESM.docx]

Supplementary Materials for

**Multidimensional tumor-blood profiling uncovers systemic lymphocyte-monocyte imbalance in pituitary neuroendocrine tumors**

Yuting Dai^1,2,#^, Shaojian Lin^1,3,#^, Junchen Wu^1,#^, Shuangshuang Yang^2,#^, Yang Lu^1^, Xiaobin Wang^1,4^, Jun Li^1^, Linfeng Zhao^5,6^, Desheng Chen^1^, Bo Zhang^1^, Yijun Cheng^1^, Hong Yao^1^, Fan Zhang^2^, Min Xu^2^, Qiang Wang^2^, Xiaojing Lin^2^, Kunjin Chen^2^, Zhen Tian^2^, Xingyan Liu^2^, Pascal Roy^7^, Hai Fang^2^, Gang Lv^2^, Tong Yin^2^, Yun Tan^2^, Bo Jiao^2^, Shengyue Wang^2^, Li Xue^1^, Youqiong Ye^5,8,*^, Saijuan Chen^2,*^, Zhe Bao Wu^1,3,5,*^

^#^ These authors contribute equally to this work

Correspondence to: youqiong.ye@shsmu.edu.cn (Youqiong Ye), sjchen@stn.sh.cn (Saijuan Chen), zhebaowu@aliyun.com (Zhe Bao Wu).

**This PDF file includes:**

Supplementary Fig. 1 to 7

**Other Supplementary Materials for this manuscript include the following:**

Supplementary Tables 1 to 8

## Supplementary Fig. 1

**
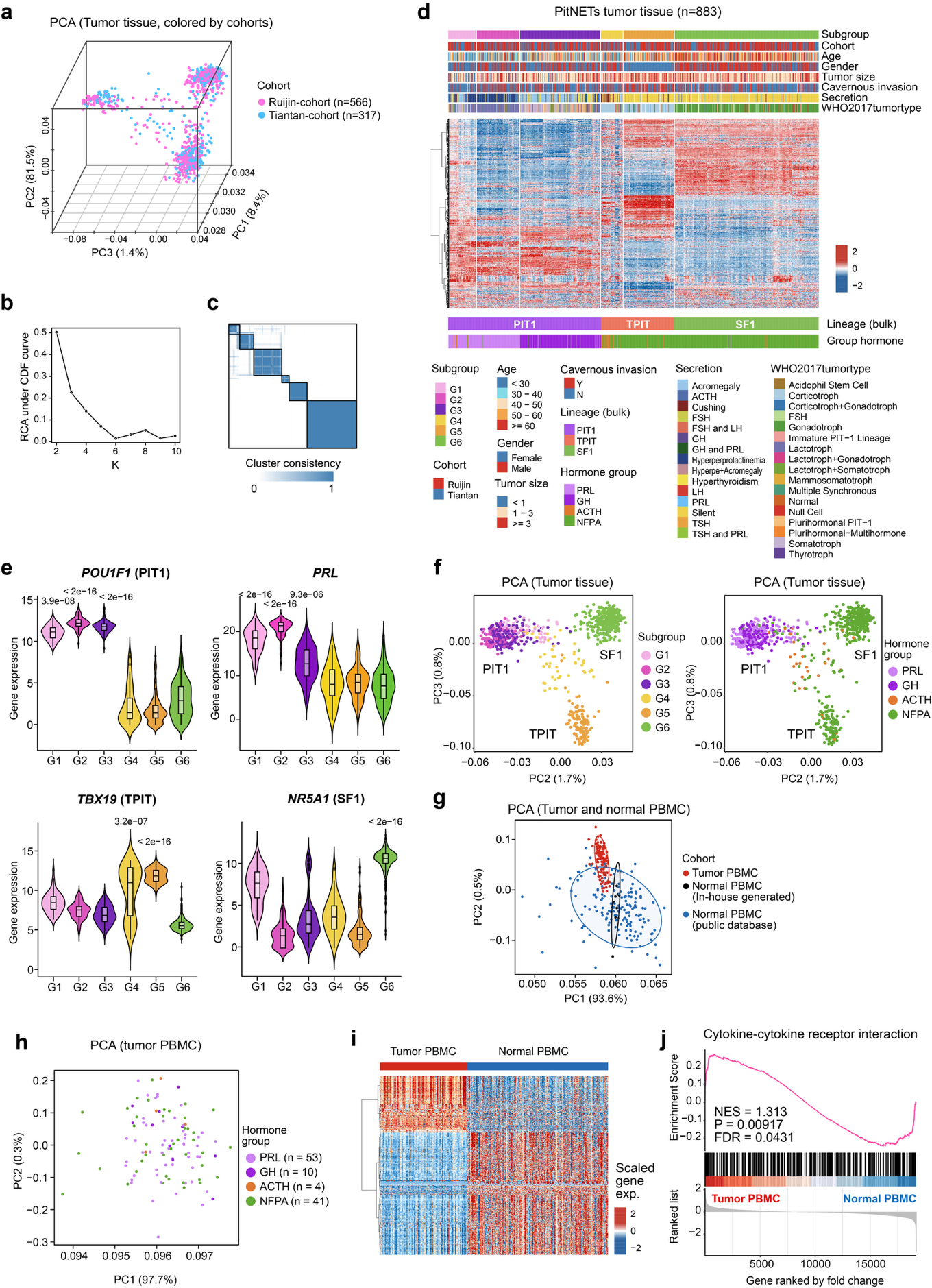
**

**Supplementary Fig. 1. Study design overview, subgroup identification, and pathway hallmarks in PitNETs, related to Fig. 1**.

**a**. Principal Component Analysis (PCA) using protein-coding genes in PitNET tumor tissues from two cohorts (Ruijin cohort and Tiantan cohort). Each point corresponds to a sample. Batch effects were adjusted according to the data source. The % value indicates the explained variance. **b, c**. Optimal cluster number determination (**b**) and consensus cluster matrix (**c**) generated using the ConsensusClusterPlus^1^ R package. **d**. Heatmap depicting gene expression patterns and clinical characteristics of PitNET tissue samples spanning three lineages and four hormone-defined subgroups. Columns represent individual patients, and rows indicate gene expression or clinical features. The top 5% variance genes were used for illustration. **e**. Violin plots illustrating expression levels of lineage-specific transcription factors (*POU1F1*, *TBX19*, *NR5A1*) across the six subgroups in PitNET tissue samples. **f**. PCA plot highlighting the six subgroups (G1-G6), the four hormone groups (PRL, GH, ACTH, clinical NFPA), and three major lineages (PIT1, TPIT, SF1) in PitNET tissue samples. Each dot represents one patient. **g**. PCA plot using protein-coding gene expression in PitNET PBMCs versus normal PBMCs. Nine in-house–generated normal PBMC samples are colored in black. **h**. PCA plot highlighting the four hormone groups. Each dot represents one patient. PCA was calculated using the 19,353 protein-coding genes. **i**. Heatmap of upregulated and downregulated genes in PitNET PBMCs compared with normal PBMCs. **j**. Gene Set Enrichment Analysis (GSEA) plot of cytokine-cytokine receptor interaction pathways in PitNET PBMCs versus normal PBMCs. Genes are ranked by fold change. The normalized enrichment score (NES), *P*-value, and false discovery rate (FDR) are labeled. *P*-values were calculated using GSEA. Two-sided *P*-values were used.

## Supplementary Fig. 2


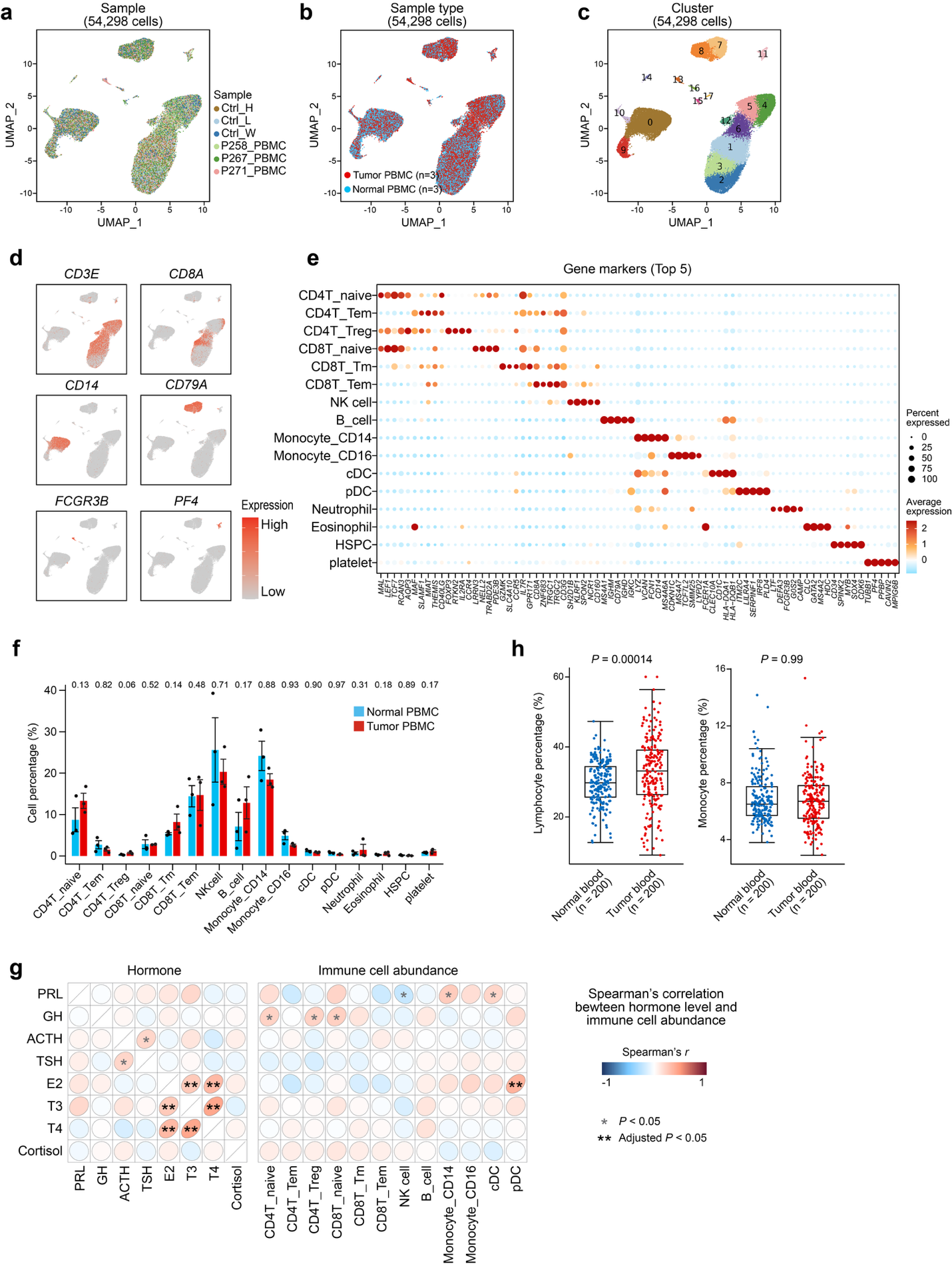


**Supplementary Fig. 2. Immune profiling of PitNET and normal PBMCs based on scRNA-seq, bulk RNA-seq and blood routine examination, related to Fig. 2.**

**a, b, c**. Uniform Manifold Approximation and Projection (UMAP) plots of single-cell RNA-seq data for 3 PitNET PBMCs and 3 normal PBMCs (Total 54,298 cells), color-coded by sample name (**a**), sample types (**b**) and clusters (**c**). **d**, UMAP plot showing cells colored according to the expression of *CD3E*, *CD8A*, *CD79A*, *CD14*, *FCGR3B*, *PF4*, respectively. **e.** Normalized expression levels and expression percentages of cell type-specific genes (top 5) across different cell types in PitNET PBMCs and normal PBMCs. **f.** Barplot comparing the percentages of all cell types between PitNET PBMCs (n = 3) and normal PBMCs (n = 3), with error bars representing the mean ± SE. *P*-values were calculated using the Student's *t-test* (one-sided). **g**. Spearman's correlation coefficients linking hormone levels with estimated PBMC cell abundances in 108 PitNET PBMC bulk RNA-seq. Red represents positive correlation and blue represent negative correlation. **h.** Boxplot comparing lymphocyte percentage (left panel) and monocyte percentage (right panel) from blood routine examination data between PitNET patients (n = 200) and healthy individuals (n = 200). *P*-values were calculated using the Mann-Whitney U test. Two-sided *P*-values were used.

## Supplementary Fig. 3


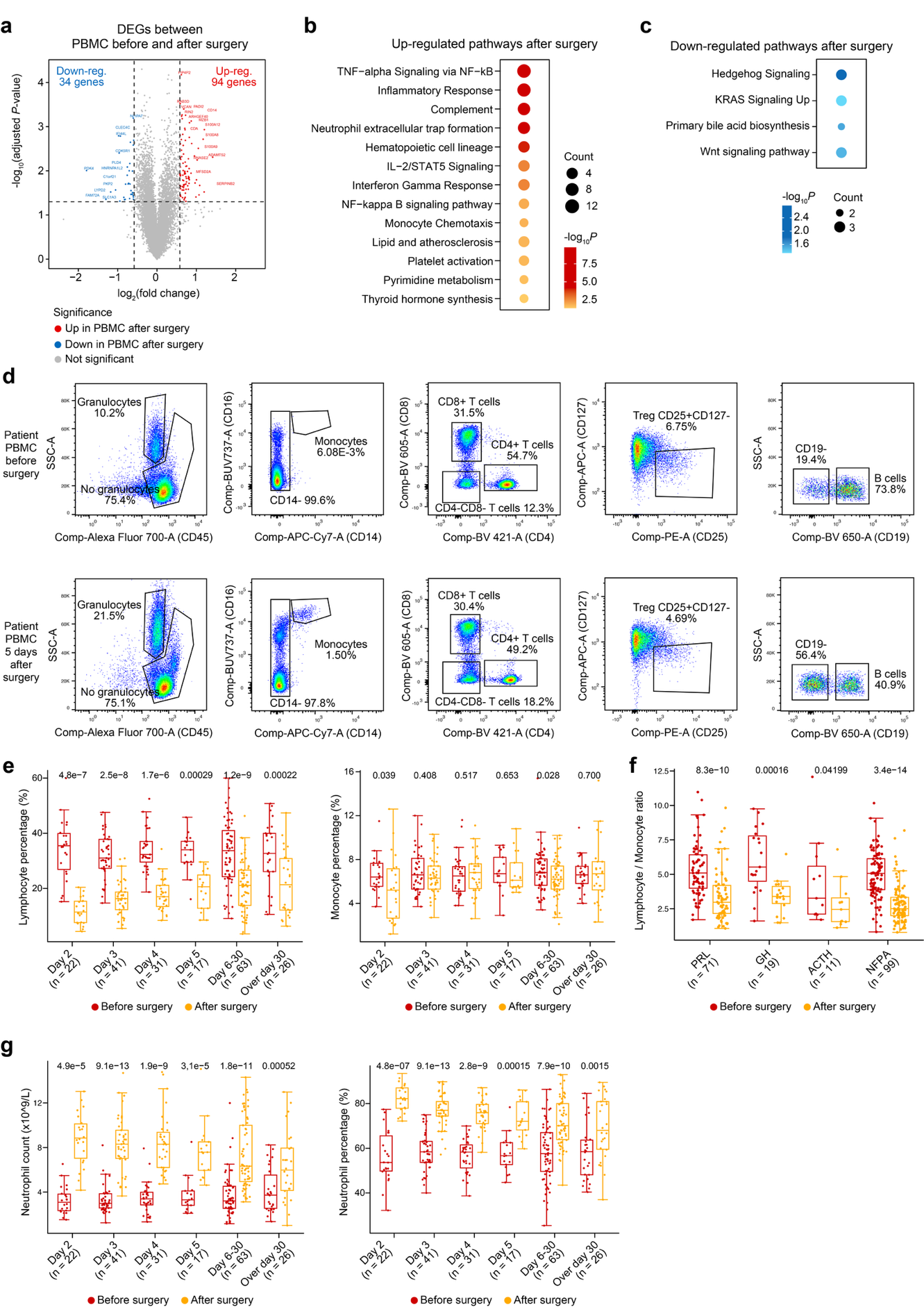


**Supplementary Fig. 3. Differential gene expression and pathway enrichment in PitNET PBMCs following surgery, related to Fig. 3.**

**a.** Volcano plot depicting differentially expressed genes (DEGs) between before and five days after surgery PBMCs from PitNET patients. In total, 34 genes were significantly downregulated (blue) and 94 were significantly upregulated (red). Each dot represents a single gene. **b**. Dot plot summarizing pathways enriched among the genes upregulated after surgery. Dot size corresponds to the number of genes, and color indicates significance (-log_10_*P*). **c.** Dot plot summarizing pathways enriched among the genes downregulated after surgery. Dot size corresponds to the number of genes, and color indicates significance (-log_10_*P*). **d.** Flow cytometry gating strategy used to quantify the proportions of granulocytes, monocytes, CD4^+^ T cells, CD8^+^ T cells, Tregs (CD25^+^CD127^–^), and B cells in fresh PBMC samples collected from PitNET patients before and five days after surgery. **e.** Box plots illustrating lymphocyte and monocyte percentages from blood routine examination in 200 PitNET patients, measured preoperatively and at multiple time points following surgery. *P*-values were calculated using the paired Mann-Whitney U test. Two-sided *P*-values were used. **f**. Boxplots comparing the lymphocyte-to-monocyte ratio in the 200 PitNET patients (by hormone groups) across preoperative and postoperative time points. *P*-values were calculated using the paired Mann-Whitney U test. Two-sided *P*-values were used. **g.** Box plots illustrating neutrophil count and percentage from blood routine examination in 200 PitNET patients, measured preoperatively and at multiple time points following surgery. *P*-values were calculated using the paired Mann-Whitney U test. Two-sided *P*-values were used.

## Supplementary Fig. 4


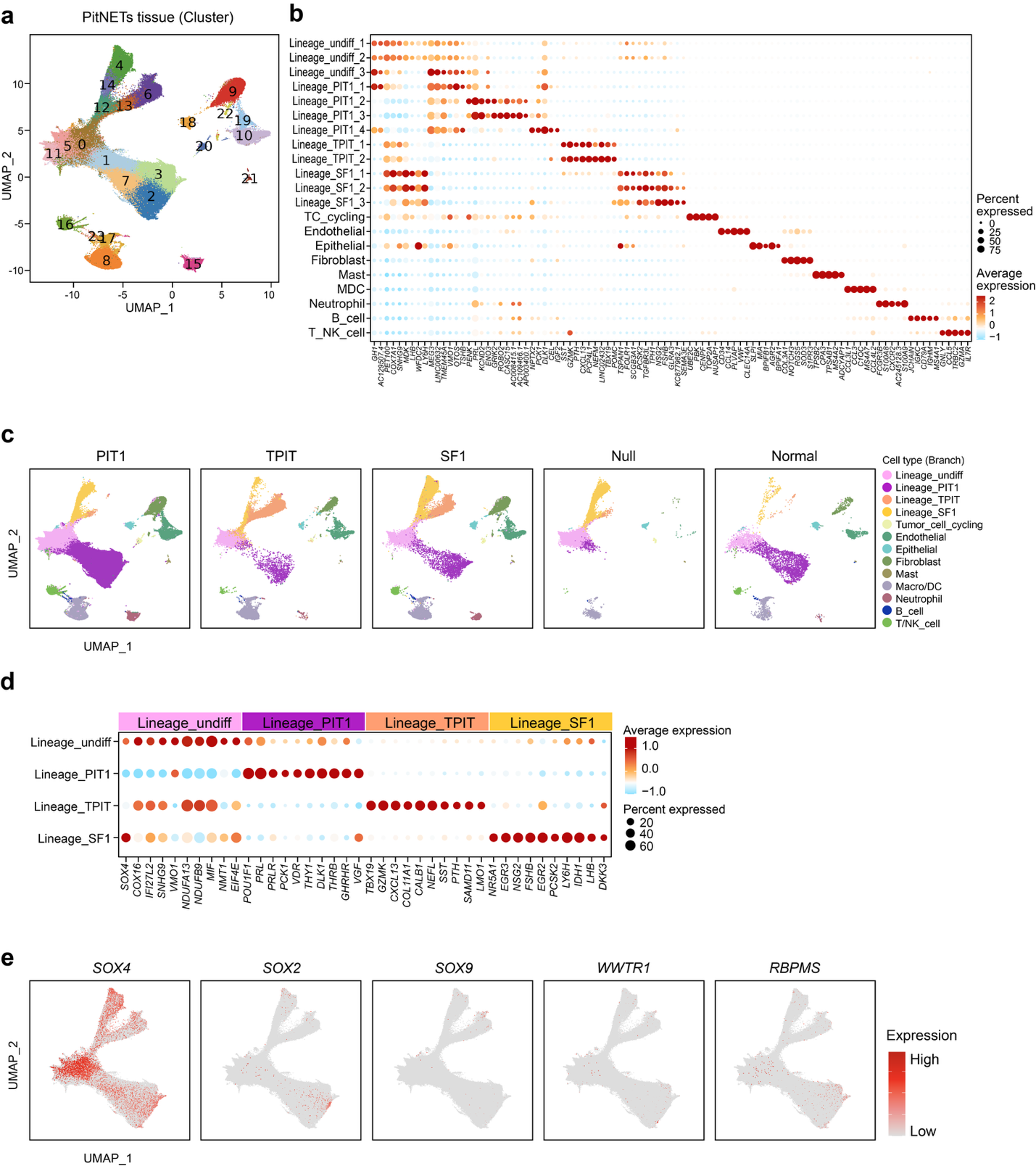


**Supplementary Fig. 4. Single-cell analysis of PitNET tissue, related to Fig. 4.**

**a.** UMAP plot showing clustering of PitNET tissue cells. Each color represents a distinct cluster. **b.** Dot plot depicting the expression patterns of marker genes across PitNET cell clusters. Dot size indicates the percentage of cells expressing a given gene, while color intensity reflects the average expression level. **c.** UMAP plots illustrating the distribution of different PitNET lineages (PIT1-lineage, TPIT-lineage, SF1-lineage, Null-cell lineage) and normal pituitary. **d**. Dot plot depicting the expression patterns of marker genes across PitNET cell lineages. Dot size indicates the percentage of cells expressing a given gene, while color intensity reflects the average expression level. **e.** UMAP plot showing cells colored according to the expression of *SOX4*, *SOX2*, *SOX9*, *WWTR1*, *RBPMS*, respectively.

## Supplementary Fig. 5


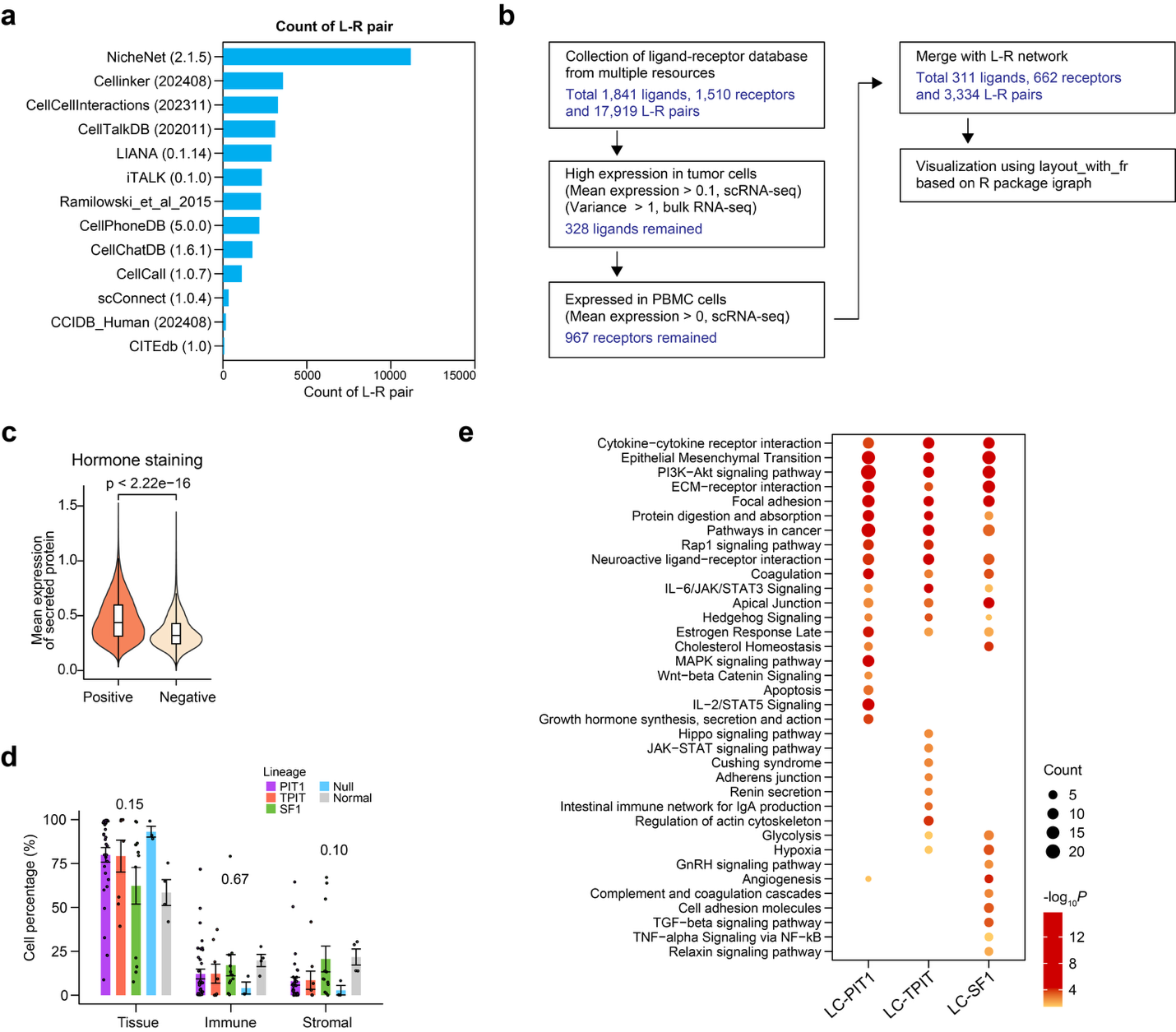


**Supplementary Fig. 5. Collection and analysis of ligand-receptor (L-R) interactions in PitNETs, related to Fig. 5.**

**a.** Bar chart displaying the number of L-R pairs aggregated from multiple cell–cell interaction tools and databases, including NicheNet, Cellinker, CellCellInteractions, CellTalkDB, and others. **b.** Schematic workflow summarizing the filtering steps and construction of the final L–R interaction network, integrating both scRNA-seq and bulk transcriptome data. **c.** Violin plots comparing the mean expression of secreted proteins in hormone-staining positive and negative PitNETs tumor tissues. *P*-values were calculated using the Mann-Whitney U test. Two-sided *P*-values were used. **d.** Barplot showing the proportions of PIT1, TPIT, SF1, and Null lineage cells within tissue, immune, and stromal compartments. Statistical significance values are indicated above each compartment group. *P*-values were calculated using ANOVA. **e.** Dotplot displaying enriched pathways for ligand cluster 1 (LC-PIT1), ligand cluster 2 (LC-TPIT), and ligand cluster 3 (LC-SF1). Each dot represents an enriched pathway, with color intensity corresponding to the statistical significance (-log_10_*P*) and dot size representing the number of associated genes.

## Supplementary Fig. 6


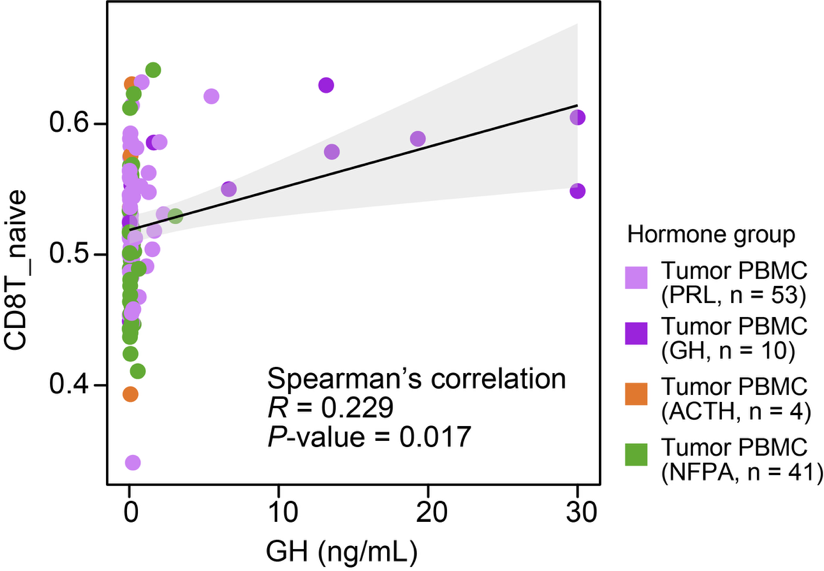


**Supplementary Fig. 6.**  **Tumor‑blood ligand-receptor crosstalk, related to Fig. 6.**

Correlation between serum GH level and the estimated PBMC CD8T_naive abundance levels. Each dot represents one patient. Spearman's correlation coefficient and *P*-value was calculated. Two-sided *P*-value was used.

## Supplementary Fig. 7


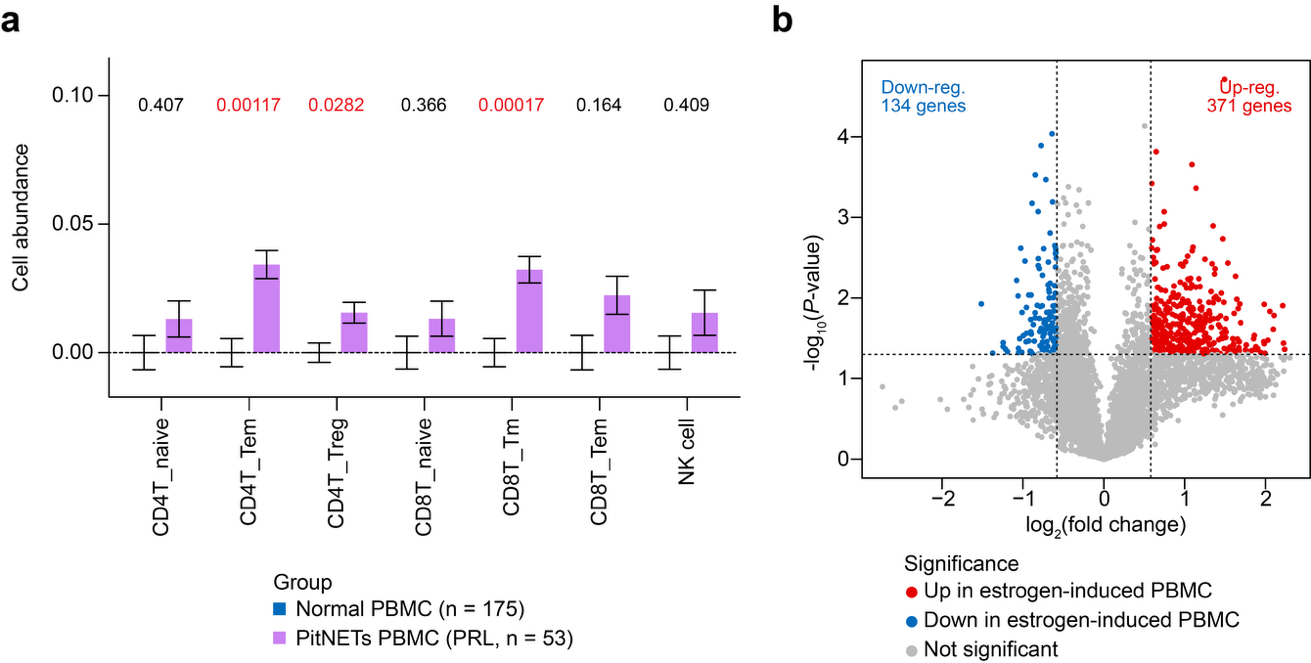


**Supplementary Fig. 7. Blood cell estimations and differential gene expression in estrogen-induced rat models, related to Fig. 7.**

**a**. Barplot comparing the estimated cell type abundance between normal PBMCs and PRL-PitNETs PBMCs bulk RNA-seq. *P*-values were calculated using the Mann-Whitney U test. Two-sided P-values were used. **b**. Volcano plot illustrating differentially expressed genes in estrogen-induced rat PBMCs compared to control PBMCs. A total of 134 genes were significantly downregulated (blue) and 371 significantly upregulated (red). Each dot represents a single gene.

## Reference

1. Wilkerson, M.D. & Hayes, D.N. ConsensusClusterPlus: a class discovery tool with confidence assessments and item tracking. *Bioinformatics* **26**, 1572-3 (2010).
